# Dissociable Effects of Urgency and Evidence Accumulation during Reaching Revealed by Dynamic Multisensory Integration

**DOI:** 10.1523/ENEURO.0262-24.2024

**Published:** 2024-12-04

**Authors:** Anne H. Hoffmann, Frédéric Crevecoeur

**Affiliations:** ^1^Institute of Information and Communication Technologies, Electronics and Applied Mathematics (ICTEAM), Université Catholique de Louvain, Louvain-la-Neuve 1348, Belgium; ^2^Institute of Neuroscience (IoNS), Université Catholique de Louvain, Brussels 1200, Belgium; ^3^WEL Research Institute, Wavre 1300, Belgium

**Keywords:** evidence accumulation, multisensory, optimal feedback control, reaching control, state estimation, urgency

## Abstract

When making perceptual decisions, humans combine information across sensory modalities dependent on their respective uncertainties. However, it remains unknown how the brain integrates multisensory feedback during movement and which factors besides sensory uncertainty influence sensory contributions. We performed two reaching experiments on healthy adults to investigate whether movement corrections to combined visual and mechanical perturbations scale with visual uncertainty. To describe the dynamics of multimodal feedback responses, we further varied movement time and visual feedback duration during the movement. The results of our first experiment show that the contribution of visual feedback decreased with uncertainty. Additionally, we observed a transient phase during which visual feedback responses were stronger during faster movements. In a follow-up experiment, we found that the contribution of vision increased more quickly during slow movements when we presented the visual feedback for a longer time. Muscle activity corresponding to these visual responses exhibited modulations with sensory uncertainty and movement speed ca. 100 ms following the onset of the visual feedback. Using an optimal feedback control model, we show that the increased response to visual feedback during fast movements can be explained by an urgency-dependent increase in control gains. Further, the fact that a longer viewing duration increased the visual contributions suggests that the brain accumulates sensory information over time to estimate the state of the arm during reaching. Our results provide additional evidence concerning the link between reaching control and decision-making, both of which appear to be influenced by sensory evidence accumulation and response urgency.

## Significance Statement

The time course of multisensory integration during movement, along with the factors influencing this process, still requires further investigation. Here, we tested how visual uncertainty, movement speed, and visual feedback duration influence reach corrections to combined visual and mechanical perturbations. Using an optimal feedback control model, we illustrate that the time course of multimodal corrections follows the predictions of a Kalman filter which continuously weighs sensory feedback and internal predictions according to their reliability. Importantly, we further show that changes in movement speed led to urgency-dependent modulations of control gains. Our results corroborate previous research linking motor control and decision-making by highlighting that multisensory feedback responses depend on evidence accumulation and response urgency in a similar way as decision-making processes.

## Introduction

Studies on perceptual judgments and decision-making have suggested that the brain minimizes uncertainty by optimally combining cues across senses according to their respective reliability (Van Beers et al., 1996; Ernst and Banks, 2002; Alais and Burr, 2004). However, natural behavior often requires to make similar decisions during movement. For instance, when we reach for an object, the brain needs to decide how much to rely on vision and proprioception to guide the arm to the desired location. Indeed, previous work has illustrated direct links between perceptual decision-making and motor control (Selen et al., 2012; Wolpert and Landy, 2012) and suggested that these processes may rely on a common brain network (Thura et al., 2022). Thus, to understand the dynamics of multisensory perception and decision-making, it is important to investigate these processes during movement control.

Seminal studies on visual feedback responses during movement have documented how changes in reach end points, and the modulation of the kinematics of corrective responses to visual perturbations, scaled with visual uncertainty and prior estimates (Körding and Wolpert, 2004; Tassinari et al., 2006; Izawa and Shadmehr, 2008). Izawa and Shadmehr (2008) interpreted their results as indicative of a continuously evolving estimate of the target state, as expected assuming the presence of a state estimator similar to a Kalman filter. In more recent work, the same model has been suggested to explain the combination of proprioceptive and visual feedback in response to combined disturbances during human postural control and reaching tasks (Crevecoeur et al., 2016; Kasuga et al., 2022) but without considering the dependency of these responses on sensory uncertainty. Here we combined these two approaches to investigate whether visual uncertainty affects feedback responses to perturbation loads during reaching.

We performed two experiments in which we varied the temporal parameters of movement execution and of the visual feedback presentation to characterize the dynamical properties of the integration process. In Experiment 1, we instructed participants to perform fast and slow movements by varying the time allowed to reach the target. Thus, by setting different timing constraints for the same movement and perturbations, we effectively manipulated the urgency to respond to the applied perturbation. Similar manipulations of response urgency by setting different timing constraints have been used in the field of motor control (Thobois et al., 2007; Crevecoeur et al., 2013; Poscente et al., 2021), as well as decision-making (Reddi and Carpenter, 2000; Thura et al., 2012; Murphy et al., 2016; Stanford and Salinas, 2021). In Experiment 2 we selectively increased the visual feedback presentation time during slow movements to assess whether this increased the contribution of vision. An optimal observer (Kalman filter) iteratively combines internal priors and sensory feedback over time. As a consequence, following a perturbation, the estimated state gradually converges toward the new state (Izawa and Shadmehr, 2008). The rate of this convergence depends on the sensory uncertainty in a similar way as the drift rate of sensory evidence accumulation during perceptual decision-making (Ratcliff, 1978).

Motor control theories based on stochastic optimal control (Todorov and Jordan, 2002) make clear predictions about the expected results. On the one hand, the control gains, with which the system responds to sensory errors, are known to be tuned to both the dynamics of the environment (Maurus et al., 2023) and the urgency to respond to a perturbation (Oostwoud Wijdenes et al., 2011; Crevecoeur et al., 2013; Dimitriou et al., 2013; Poscente et al., 2021). On the other hand, considering additive noise as a first approximation, the dynamics of state estimation only depend on the statistics of the noise disturbances, and not on the urgency. Thus, in principle, there should be a transient effect of movement time on the modulation of feedback responses attributable to the control gains, while the estimation following visual errors should only depend on the sensory information available to the brain.

Our results were remarkably similar to the model predictions. First, assuming the presence of a Kalman filter could explain uncertainty-dependent modulations in feedback responses to multimodal perturbations. Second, simulating urgency-related modulation of feedback responses reproduced a transient increase in the visual feedback response for similar amounts of visual information that was observed in the data. Third, we observed that the contribution of visual feedback increased with viewing duration, which bared an obvious resemblance to a process of evidence accumulation over time. In all, we found both an effect of urgency and evidence accumulation during multimodal perturbation responses and suggest that these factors are separable computational operations underlying both movement control and decision-making.

## Materials and Methods

### Participants

This study is based on data collected from 32 healthy young adults aged 19–35. Sixteen (11 females) participants took part in Experiment 1 and sixteen (10 females) in Experiment 2. Handedness was assessed using the Edinburgh Handedness Inventory (Oldfield, 1971), and all participants reported to be right-handed. All participants had normal or corrected to normal vision, and none of them indicated suffering from a neurological or motor disorder. Prior to participating in the experiment, participants were informed about the experimental procedure and gave written consent. All procedures were approved by the ethics committee at the host institution (*Comité d’Éthique Hospitalo-Facultaire*, UCLouvain). In total the experiment took 3 h including information and preparation of the participant. All participants received a small financial compensation for their time.

### Apparatus and general task procedure

**Figure 1.**
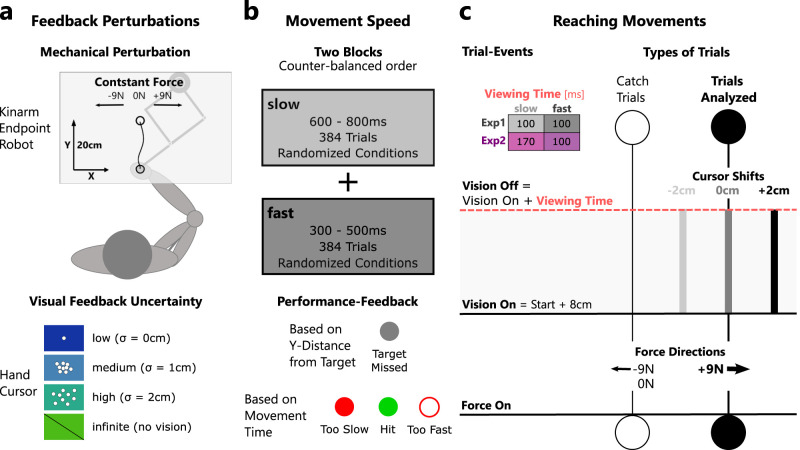
Experimental design. ***a***, Feedback perturbations: Top panel, participants performed 20 cm right arm reaching movements holding the handle of a robotic manipulandum. At movement onset, a ±9 N constant load or no load (0 N) was applied to perturb the arm orthogonal to the movement direction. Bottom panel, Visual feedback was presented as a hand cursor and varied in uncertainty. In the condition of low uncertainty, a single hand cursor was presented (*σ* = 0 cm), in conditions of medium and high uncertainty a cloud of 25 cursors was presented (*σ* = 1 cm, *σ* = 2 cm). In a fourth condition, no visual feedback was presented (infinite). ***b***, Movement speed: Top panel, the experiment consisted of two movement speed conditions that were applied in sessions of 384 trials each. The order of movement speed sessions was counterbalanced across participants. Movement speed was manipulated by imposing different timing constraints on participants’ movements. In the slow session, movements were counted as hits if the movement time was between 600 and 800 ms and in the fast session between 300 and 500 ms. Within each movement speed session, visual uncertainty conditions, force directions, and cursor shifts were applied in a random order. Bottom panel, Participants received feedback about the timing and length of their movement via a change in target color at the end of each movement. ***c***, Reaching movements, force perturbations were turned on when the movement was initiated and remained on during the movement and the stabilization phase. Visual feedback was presented for 100 ms (Exp. 1 fast and slow, Exp. 2 fast) or 170 ms (Exp. 2 slow) once the hand crossed a distance of 8 cm from the start target. Cursor shifts were only applied during trials with rightward 9 N perturbations and the analyses focused on these trials only.

Both experiments were conducted using a KINARM Endpoint robotic device (KINARM). The task paradigm was developed using Matlab's Simulink and Stateflow toolboxes (Matlab 2015, MathWorks). During the experiment, participants held the right handle of the KINARM robot and performed 20 cm forward reaching movements with their right arm (Fig. 1*a*). The start and goal targets were displayed as gray circles with a radius of 1.2 cm and were projected into the plane of the movement using a monitor–mirror setup. The start target was located 8 cm from the bottom of the screen and 9 cm to the right of the body midline, and the goal target was located 20 cm straight ahead from the start. Direct view of participants’ hands was blocked throughout the experiment, but their hand position was indicated on the screen between trials using a 0.5-cm-radius white cursor. During the movement, the visual feedback changed as explained in more detail below. To start each movement, participants were instructed to move the cursor into the start target. Upon entry, the start target changed color from gray to blue and a waiting time interval of 2–4 s (drawn from a uniform random distribution) was generated. The cue to initiate the movement was given by changing the color of the goal target to blue as well. In 75% of all trials, a rightward constant load of 9N was applied to the hand as soon as it left the start target and remained on for the entirety of the trial (Fig. 1*c*). During the remaining 25% of trials, participants experienced either no force or a leftward 9N force. The applied forces were ramped up and down over a period of 5 ms.

During the initial and final phases of the movement, no visual feedback of the hand position was shown to participants. However, when the hand crossed a threshold of 8 cm straight ahead from the start target, visual feedback of the hand position was flashed on the screen for 100 or 170 ms (Fig. 1*c*; see details about Exp. 1 and 2 below). To manipulate visual uncertainty, we presented either a single hand cursor, or a cloud of cursors of increasing spread, or no visual feedback about the hand location (Fig. 1*a*). We adapted this experimental manipulation from a previous study (Körding and Wolpert, 2004), but similar techniques have been used elsewhere (Ferrari and Noppeney, 2021; Tsay et al., 2021). In the single cursor condition, the hand position was indicated by a white cursor of 0.3 cm radius (low visual uncertainty). The cursor clouds were composed of 25 dots of 0.2 cm radius. The position of each cursor was drawn randomly from a normal distribution centered on the hand position with a standard deviation of 1 cm (medium uncertainty) or 2 cm (high uncertainty) in *x*- and *y*-dimensions. The trials without visual feedback served as a control condition with theoretically infinite visual uncertainty. On 50% of trials the center of the hand cursor or cursor cloud (even if invisible) was shifted 2 cm to the left or right relative to the true location of the hand to induce discrepancies between the felt and seen hand location (cursor shift). This allowed us to quantify the influence of the visual feedback on the corrective response (see below, Movement kinematics and forces: data collection and analyses, for details). These cursor shifts were only imposed on trials with rightward force perturbations to limit the total number of trials. Consequently, our analyses focused exclusively on those trials. Trials with leftward or no force perturbation served to make the task less predictable and keep participants focused.

We instructed participants to perform straight movements to the target and to stabilize their hand there for 2 s (stabilization phase). For trials with mechanical perturbations, they were asked to compensate for the force and stabilize their hand as close as possible to the goal target. For a successful trial, participants had to cross the distance of 20 cm straight ahead from the start target within the imposed time interval (Fig. 1*b*; see details on Exp. 1 below). However, participants did not receive feedback about their movement endpoints, and trial success was not dependent on whether they landed on target in the lateral dimension of the movement. Upon movement completion, participants received feedback in the form of a color change of the goal target (Fig. 1*b*). There were four possible scenarios: if the goal target changed back to gray, it signaled that the participant had undershot the target and that they should try to execute a longer movement on the next trial (these trials were excluded from further analyses); if the goal target filled red or turned to a red outline, it meant that the participant's movement had been too slow or too fast, respectively; lastly, if the goal target changed to green, it indicated that the movement was performed correctly within the imposed time interval. To increase participants’ motivation, they gained one point for each successfully completed trial and were told to try to collect as many points as they could. Following the presentation of the feedback, the goal target was extinguished. To start the next movement, participants had to move the handle back toward the start target and the hand cursor reappeared on the screen when the handle was within a 10 cm radius of the start target. This was done to avoid that participants would notice shifts in the cursor position that were imposed during the experiment.

### Experiment 1

The goal of Experiment 1 was twofold. Firstly, we aimed to investigate the effect of visual hand feedback uncertainty on feedback corrections to a mechanical perturbation. Secondly, we aimed to study whether the response to the visual feedback additionally depended on the speed of the performed movement. During this experiment visual feedback was presented for 100 ms during the movement across two different conditions of movement speed (Fig. 1*b*,*c*).

The experiment was divided into two separate parts, one in which the movement time window was set between 300 and 500 ms (fast movement speed condition) and a second condition in which movement time was between 600 and 800 ms (slow condition; Fig. 1*b*). In each condition, movements were rewarded with a point if they were performed within the instructed time intervals. Each part was preceded by two practice blocks of 10 movements without force perturbation, followed by 20 movements with load perturbations (10 leftward and 10 rightward), so that participants could familiarize themselves with the task and the movement speed requirement. If they failed >50% of the practice movements, the training block was repeated once. The order of speed conditions was counterbalanced across participants to limit potential biases linked to the first practiced condition.

Each condition was divided into six blocks of 64 trials each. Each of these blocks consisted of 48 trials with a rightward force perturbation (four repetitions of four visual uncertainty by three cursor shift combinations), 8 trials with leftward force perturbation, and 8 trials without force (two repetitions of four possible visual uncertainty conditions). The order in which these trials appeared was completely randomized. When needed, participants were allowed to take short breaks between blocks. Usually a longer break of ∼5 min was inserted between the first and second part of the experiment. In total each participant performed 768 trials and the experiment lasts ∼2.5 h in total. At the end of the experiment, each participant was asked whether they noticed something during the experiment, but none of them reported having noticed the cursor shifts.

### Experiment 2

Experiment 2 was performed as a follow-up experiment to investigate whether the differences between fast and slow movements observed in Experiment 1 could be at least partly explained by the difference in distance during which the visual feedback was visible. Put differently, we were interested to see whether increasing the duration of the visual feedback during slow movements would increase the visual contribution to movement corrections. In Experiment 1 we imposed a fixed 100 ms time interval during which the visual feedback was shown. Given the different movement speeds, this resulted in a difference of movement distance during which visual feedback was available in the fast and slow conditions. Analyses revealed that in Experiment 1 visual feedback was present on average (mean ± SD) for 5.7 cm (±0.9 cm) in the fast condition and for 3.2 cm (±0.7 cm) in the slow condition. To account for the potential influence of this difference on our result of Experiment 1, we designed a second experiment in which we matched the distance traveled with visual feedback between the fast and slow movement condition based on the difference in group average velocity. To achieve this, we extended the visual feedback viewing time in the slow condition to 170 ms (Fig. 1*c*). This resulted in an average distance traveled with visual feedback of 5.6 cm (±0.9 cm) in the fast and 5.3 cm (±1.2 cm) in the slow condition. Importantly, the distance at which the visual feedback was turned on remained at 8 cm from the start target in both movement speed conditions. All other experimental procedures also remained identical to Experiment 1.

### Movement kinematics and forces: data collection and analyses

During the experiment, we recorded movement kinematics as well as forces at a sampling rate of 1 kHz using KINARMS's Dexterit-E software (version 3.9). We measured the interaction forces between the hand and the robot using an ATI force sensor located at the top of the KINARM handle. These measured forces were mapped into the *x*- and *y*-coordinate system of the experimental workspace using the Dexterit-E software. As the lateral motion of the handle was not constrained during the movement, the recorded forces correlate with the acceleration of the movement. Importantly, these recordings reflect both the forces applied by participants on the handle and mechanical interactions induced by the arm's mechanical impedance and the robot dynamics. Consequently, the absolute force magnitude does not correspond to the force participants applied on the handle. However, as we can assume that these mechanical interactions are constant throughout the experiment, we interpreted modulations in the reaction force as an approximation of the change in participants’ feedback responses. Preprocessing of the data was performed using custom-written MATLAB scripts (MATLAB 2021a, MathWorks). The recorded positions were filtered using a low-pass, fourth-order, dual-pass Butterworth filter with a cutoff frequency of 50 Hz. Hand velocities were derived from the raw position data using a fourth-order central difference approximation and passed through the same filter.

To quantify the influence of the visual feedback on movement corrections, we extracted the lateral hand position during rightward perturbation trials at different time points during the movement following the onset of the visual feedback. In particular, we extracted this information for each participant at 150, 300, 500, 700, and 900 ms following the onset of visual hand feedback. It should be noted that each of these time points lay outside of the 100 ms time window during which vision was presented in Experiment 1. We chose these specific time points based on visual inspection of the data as they track well the development of the influence of the visual feedback. Next, we computed the regression slope between the cursor shift that was applied and the lateral position of the hand relative to the center of the target at each time point. This regression slope served as a quantification of how much participants relied on the visual feedback. A slope of 0 signified that there was no shift in the lateral position of the movement relative to the cursor shift and hence no influence of the visual feedback. On the contrary, a slope of −1 meant that participants fully compensated for the 2 cm lateral cursor shift. Lastly, we computed the average regression slopes across all participants and compared these values across the conditions of visual uncertainties and movement speeds.

Additionally, we computed the variability of positions at the same five time points during the movement (150, 300, 500, 700, and 900 ms) based on the two-dimensional dispersion ellipses of *x*- and *y*-positions for each visual uncertainty condition using singular value decomposition and defined the variability as the area of the ellipse. Finally, we looked at the lateral forces applied to compensate for the perturbation. For this analysis, we first computed the difference in average lateral forces between the rightward or leftward cursor shift condition and the no cursor shift condition for each participant separately. Next, we computed the difference of this difference (rightward cursor shift − no cursor shift) − (leftward cursor shift − no cursor shift) and extracted the maximum value of this quantity.

### Electromyography: data collection and analyses

We recorded EMG (electromyography) signals from the pectoralis major (PM) and posterior deltoid (PD) muscles in the right shoulder. These muscles act as agonists to the right- and leftward force perturbation, respectively. Muscle activity was recorded using bipolar surface electrodes (DE-2.1 EMG Sensor, Delsys) which were attached over the muscle belly. Prior to applying each electrode, we cleaned the skin underneath with cotton gauze and medical alcohol and coated the contacts of each electrode with conductive gel to enhance the signal-to-noise ratio. Depending on the signal strength of each participant, we amplified the signal by a factor of 1,000 or 10,000 (Bagnoli-8 EMG System, Delsys). All EMGs were recorded at a sampling frequency of 1 kHz.

The preprocessing and analysis of the muscle recordings was performed using custom-written MATLAB scripts (MATLAB 2021a, MathWorks). First, we aligned the EMG recordings to movement onset and bandpass filtered the signal using an eighth-order, dual-pass Butterworth filter (cutoff frequencies: [20, 250] Hz). After filtering, signals were rectified and normalized by the average activity computed based on four separate normalization blocks which were performed before and after each speed condition of the experiment. During these calibration trials, participants were presented a 2 by 2 cm square on the screen in front of them. As soon as they moved their hand inside the square, a 9 N constant force was applied to the left or right against their hand to activate one of the two muscles of interest. This force remained on for 2 s, and participants were instructed to counter the force and keep their hand inside the square on the screen. For the normalization, we extracted a 1 s recording between 0.5 and 1.5 s following the onset of the force. Next, we computed the mean rectified muscle activity across this time window for all the repetitions of each force direction. Finally, the activity measured in each muscle during all trials was divided by their corresponding calibration values.

To investigate the influence of visual uncertainty on the modulation of muscle responses, we realigned the preprocessed EMG recordings to the presentation onset of the visual feedback and computed average traces for each visual uncertainty and cursor shift condition across participants. To improve the illustration of the group-average EMG traces, we plotted a moving average with a window size of 11 samples. Next, we computed the difference between conditions with a cursor shift to the right and left to illustrate the effect of the visual uncertainty on the modulation of muscle activity with the direction of the cursor shift. For illustration of these delta EMG traces, we again plotted a moving average with a window size of 31 samples across 0–300 ms following the onset of the visual feedback. Lastly, to compare the different visual uncertainty conditions statistically, we computed the average delta EMG responses over a time window from 100 to 250 ms following the onset of the visual feedback.

### Experimental design and statistical analyses

We performed our statistical analyses using custom-written MATLAB scripts (MATLAB 2021a, MathWorks). For our main analyses, we used a four by two repeated-measures ANOVA with visual uncertainty and movement speed as within participant factors. We chose to use repeated-measures ANOVA because it allowed for a simple summary of our data and statistical results (i.e., the effect of the visual uncertainty is visible as a main effect instead of an interaction between cursor shift and visual uncertainty). For consistency, we used the same statistical test throughout all of our analyses. We assessed the main effect of visual uncertainty and movement speed as well as their interaction on the slope and the movement variability at each of the five specified time points following the onset of the visual feedback (150, 300, 500, 700, and 900 ms). Further, we analyzed the influence of visual uncertainty and movement speed on the absolute and delta lateral forces as well as the delta EMG. Lastly, we compared the slopes across Experiments 1 and 2 using a repeated-measures ANOVA with movement speed as within participant factor and experiment number as between participant factor. For each ANOVA, we report the *F* statistic, the degrees of freedom, the *p* value, and the partial eta squared as a measure of effect size (Lakens, 2013). To highlight significant mean differences in our figures, we computed post hoc pairwise comparisons with Bonferroni’s corrections. For within-participant comparisons, all statistical results with a corrected *p* value <0.005 are considered significant (Benjamin et al., 2018; Lakens et al., 2018). Additionally, results with a *p* value <0.05 are interpreted as a significant trend. For between-participants comparisons, statistical results are regarded as significant if the *p* value was below a threshold of 0.05.

### Model

We used an optimal control model to simulate the influence of visual uncertainty and movement speed on feedback corrections to combined mechanical and visual perturbations. Our model describes the translation of a point mass (*m *= 1 kg) in a plane. Such a simplified linear model has been previously used to approximate the nonlinear behavior of a multijointed arm (Izawa and Shadmehr, 2008; Nashed et al., 2012). The model includes a damping factor *G *= 0.1 Nsm^−1^, and we approximated the muscle dynamics using a first-order low-pass filter with time constant *τ* = 66 ms (Brown and Loeb, 2000). The state variables include the hand position (p) and velocity $\left(\dot{\;p}\right)$, the commanded force $\left(F_{\mathrm{Com}}\right)$ produced by the control input (u), and the external force used to simulate the mechanical perturbation $\left(F_{\mathrm{Ext}}\right)$. Additionally, we added a variable for the cursor motion (*p*_*c*_), as well as a variable to define an offset between the cursor and hand motion $\left(\;p_{\mathrm{off}}\right)$. This procedure was chosen to allow dissociating the hand from the cursor as in the experiment (cursor shift) and make the offset variable nonobservable such that any shift between cursor and hand had to be estimated (these modeling choices are not the unique way of dissociating hand and cursor position). Thus, the state vector is defined as follows:

$$x\;=\;[\;p,\dot{p},F_{\mathrm{Com}},F_{\mathrm{Ext}},p_{c},p_{\mathrm{off}}{]}^{T}.$$

Finally, we augmented the state vector with the target state variables. The continuous differential equations of the system are the same for *x*- and *y*-dimensions without interaction. For simplicity we only describe the dynamics in the *x*-dimension here corresponding to the lateral hand coordinate and verified that similar conclusions hold when the two dimensions of the plane are not independent such as with signal-dependent noise aligned with the control vector (Extended Data Figs. 8-1, 8-2):

$$\{\begin{array}{c} m\ddot{p}=-G\dot{p}+F_{\mathrm{Com}}+F_{\mathrm{Ext}}\\ \tau \;{\dot{F}}_{\mathrm{Com}}=u-F_{\mathrm{Com}}\\ {\dot{F}}_{\mathrm{Ext}}=0 \end{array}.$$

The third equation expresses that changes in the external force input are assumed to follow a step-function.

Next, the dynamics of the system were discretized using Euler integration with a time step of *δt* = 10 ms. This led to the following representation of the discrete time control system:

$$x_{t+1}=Ax_{t}+Bu_{t}+\;\xi_{t}\;,$$

$$A=\left[\begin{array}{cccccc} 1 & \delta t & 0 & 0 & 0 & 0\\ 0 & 1-\frac{G\delta t}{m} & \frac{\delta t}{m} & \frac{\delta t}{m} & 0 & 0\\ 0 & 0 & 1-\frac{\delta t}{\tau } & 0 & 0 & 0\\ 0 & 0 & 0 & 1 & 0 & 0\\ 1 & \delta t & 0 & 0 & 0 & 1\\ 0 & 0 & 0 & 0 & 0 & 1 \end{array}\right],B=\left[\begin{array}{c} 0\\ 0\\ \frac{\delta t}{\tau }\\ 0\\ 0\\ 0 \end{array}\right],$$

where $\;\xi_{t}$ is an additive multivariate Gaussian noise with zero-mean and known covariance matrix $\left(\Sigma_{m}\right)$. The last two rows of the matrices *A* and *B* correspond to the cursor position and the offset between cursor and hand, respectively. Calling $p_{c,t}$ the cursor position and $p_{\mathrm{off},t}$ the offset between hand and cursor at time t, the discrete time dynamics of these variables are, respectively, $p_{c,t+1}=p_{t+1}+p_{\mathrm{off},t}$ and $p_{\mathrm{off},t+1}=p_{\mathrm{off},t}$.

As mentioned above, we assumed observability of all state variables except the offset between hand and cursor position. Hence, the observation matrix H is defined as diag(1,1,1,1,1,0) and the feedback equation can be written as follows:

$$y_{t}=Hx_{t}+\;\omega_{t},$$

where $\omega_{t}$ is the sensory noise with covariance matrix $\Sigma_{\omega }$. We manipulated the visual uncertainty by increasing or decreasing the corresponding element in $\omega_{t}$ to simulate different amounts of noise in the feedback about the cursor position. This was done arbitrarily by multiplying or dividing $\Sigma_{\omega }$ by a factor of 10. This procedure was constrained by the experimental design in which the sensory signal was actually increased or decreased, but the factors used could not match noise statistics of visual estimates accurately, so we verified that they produced differences in slopes that were broadly consistent with the behavioral observations.

Next, we used Kalman filtering to obtain maximum-likelihood estimates of the system state at each time point. This estimator assumes an optimal combination of prior and sensory feedback which relies on internal knowledge of state-space representation matrices ( A, B, and H), control input, and noise covariance matrices. The prior is the expected value of the next state given the current estimate $\left({\hat{x}}_{t}\right)$ and the control input $\left(u_{t}\right)$ and is computed by simulating the system dynamics over one time step:

$${\hat{x}}_{t+1}^{p}=\;A{\hat{x}}_{t}+Bu_{t}.$$

The estimated state at the next time step is then computed by combining the prior with the observed feedback error weighted by the Kalman gain (K):

$${\hat{x}}_{t+1}={\hat{x}}_{t+1}^{p}+K(y_{t}-H{\hat{x}}_{t}^{p}).$$

Please note, to ensure corrective responses to the mechanical and visual perturbations, Kalman gains were computed using nonzero motor noise for the external force and the hand cursor offset. This allows the estimator to infer step changes in this variable, which is otherwise impossible if it is assumed that there is no motor noise affecting these variables. Thus, to compute the Kalman gains, we added 0.1% of the defined motor noise $\left(\Sigma_{m}\right)$ to the cursor position and 1% to the external force. For the movement simulations, the motor noise for these variables was then set back to zero in agreement with their physical properties.

We computed optimal control gains using a quadratic cost function with a penalty on position error and control:

$$J(x_{t},u_{t})=\;x_{t}^{T}Qx_{t}+\;u_{t}^{T}Ru_{t},$$

where *R* = 10^−4^ describes the cost to penalize large control commands and *Q* represents the cost applied to position errors during the 500 ms stabilization phase at the end of the simulated movements. Thus, *Q* was zero throughout the movement and applied a quadratic cost term to the difference between cursor and target position during the stabilization phase. The resultant optimal control policy is a linear function of the estimated state:

$$u_{t}=-L_{t}{\hat{x}}_{t},$$

with L representing the optimal control or feedback gains. Details about the derivation of the controller can be found elsewhere and followed standard techniques (Åström, 1970; Todorov, 2005).

To simulate mechanical perturbations, we set the *x*-component of the external force $\left(F_{x,\mathrm{Ext}}\right)$ to 9 N during a simulation run as soon as the y position of the state exceeded a distance of 0.5 cm from the start position. Additionally, we added a shift of the cursor at 8 cm from the start position by setting the offset between hand and cursor position in the *x*-dimension $\left(\;p_{x,\mathrm{off}}\right)$ to ±2 cm. We computed 25 simulation runs with movement time 400 and 700 ms for fast and slow movements, respectively. At the end of each movement, we added a 500 ms stabilization phase to mimic the experimental paradigm as closely as possible.

### Software accessibility

The Python code for these model simulations has been deposited on GitHub at https://github.com/annehoff/MultisensFBReaching and is publicly available. The code is also available as Extended Data 1.

10.1523/ENEURO.0262-24.2024.d1 Extended Data 1 Model code. Download Extended Data, ZIP file.

## Results

One of the hallmarks of optimal multisensory integration is that the contribution of each sensory cue is weighted by the inverse of its variance (reliability). Therefore, Experiment 1 aimed to investigate whether visual uncertainty also modulated online feedback responses to mechanical loads applied during reaching. Moreover, we varied movement time to test whether this influences the dynamics of corrective responses to combined force and visual perturbations.

**Figure 2.**
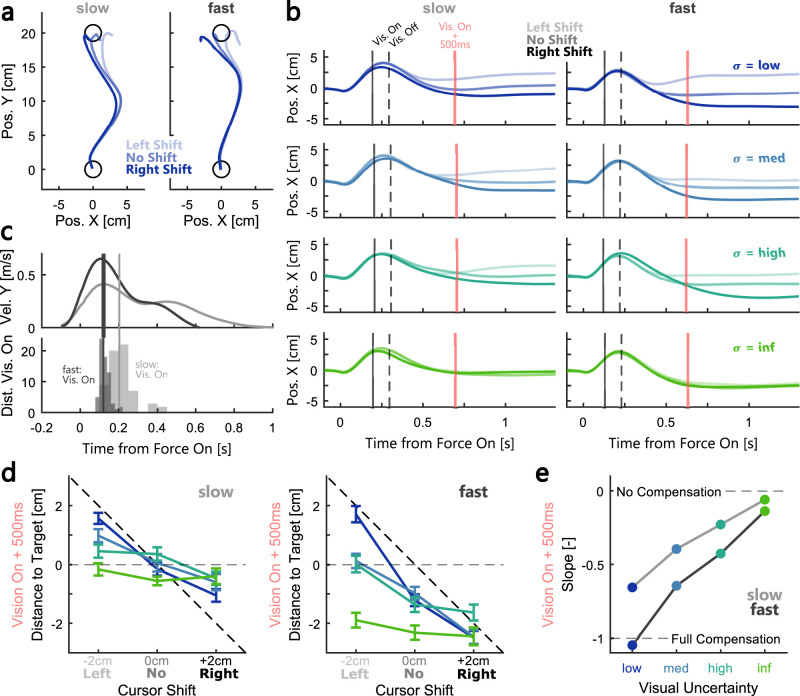
Example participant behavior, quantification of visual feedback influence as the slope of lateral hand positions relative to the target. ***a***, Average hand trajectories during low visual uncertainty for one example participant in Experiment 1. Trajectories during slow movements are shown on the left panel, those during fast movement on the right panel. Shades of blue represent the three cursor shift conditions for trials with low visual uncertainty. ***b***, Average lateral position over time for the same example participant. The left column represents averages across slow movements (visual uncertainty low—inf are shown in top to bottom panel). The right column represents averages across fast movements. Different shades of the same color represent different cursor shifts (light, left; medium, no; dark, right). The vertical black lines represent the median onset (full line) and offset (dashed line) times of the visual feedback, and the red line marks the median value of the time point 500 ms after vision onset. ***c***, Top, Average forward velocities during fast (dark gray line) and slow (light gray line) movements without cursor shift and with low visual uncertainty for the same example participant. Bottom, Histograms of the onset times of the visual feedback relative to force onset for the same participant. The slow condition is shown in light gray and the fast in dark gray. Vertical lines represent the median onset time during slow (dashed) and fast (full) conditions. ***d***, Left, Average lateral positions extracted 500 ms after vision onset (see red lines in panel ***b***) relative to the target center as a function of the cursor shift during slow movements. Blues and greens represent the different visual uncertainties. Right, Same as left but for fast movements. ***e***, Average slopes between cursor shift and lateral hand position as a function of visual uncertainty and movement speed (slow, light gray; fast, dark gray) for the same example participant computed at 500 ms after vision onset.

Figure 2*a* displays the average trajectories during rightward perturbations for one exemplar participant in Experiment 1. The different directions of the cursor shift are shown in different shades of blue. Similarly, panel b shows the average lateral positions for the same participant. The black vertical lines indicate the median onset and offset time of the visual feedback for this participant. In trials in which visual feedback was presented during the movement, we can observe a clear divergence of the lateral positions in correspondence with the direction of the cursor shift. Specifically, when the cursor was shifted 2 cm to the right relative to the hand coordinate, the participant's corrective response increased. Conversely, when the cursor was shifted 2 cm to the left, closer to the midline of the movement, the correction was reduced. Thus, the visual feedback clearly influenced the feedback response to the force perturbation.

The top panel of Figure 2*c* shows the average forward velocity during fast (dark gray line) and slow (light gray line) movements for the same example participant, while the bottom panel depicts the distribution of the onset times of the visual feedback. The vertical lines indicate the median onset time in each movement speed condition for this participant. It is visible that the onset of the visual feedback occurred after peak velocity in both conditions.

Next, to compare the contribution of the visual feedback across different levels of uncertainty and movement speed, we extracted the lateral positions at 150, 300, 500, 700, and 900 ms after the onset of the visual feedback. Figure 2*d* shows the lateral positions at 500 ms as a function of the cursor shift for the same exemplar participant during slow (left panel) and fast (right panel) movements. We computed the slope across the cursor shifts and plotted these values as a function of the visual uncertainty (Fig. 2*e*). A slope of 0 indicates that the movement corrections did not differ depending on the cursor shift, whereas a slope of −1 signifies that the participant compensated fully for the shift of the cursor or the expected value of the cursor cloud. Intermediate values of the slope indicate a partial correction for the visual shift. Figure 2*e* clearly illustrates that the contribution of the visual feedback to the movement correction decreased with visual uncertainty in both movement speed conditions.

**Figure 3.**
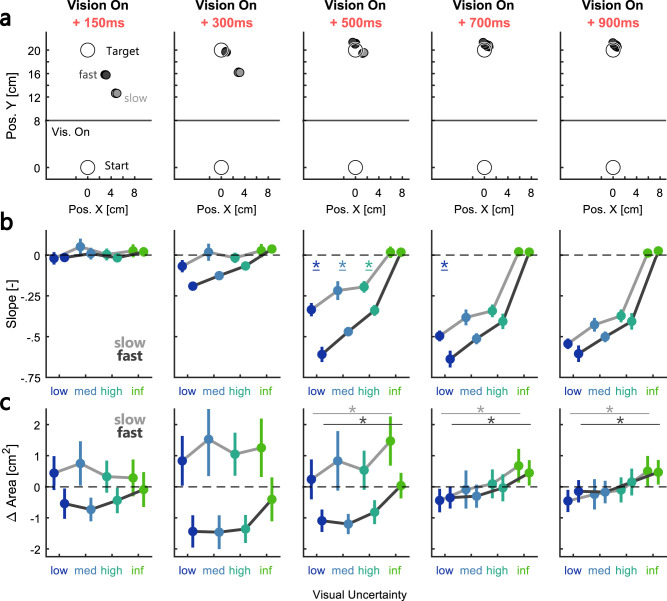
Gradual influence of visual feedback during movement varied with visual uncertainty and movement speed. ***a***, Group average location of the hand relative to the target during the two movement speed sessions at different time points relative to vision onset. Hand positions were averaged across trials without cursor shift and light gray circles represent slow and dark gray circles fast movements, respectively. See Extended Data Figure 3-1 for an additional analysis of the offset in lateral hand positions relative to the target midline across the different time points. ***b***, Group average slopes computed at different time points after the onset of the visual feedback as a function of visual uncertainty. The slopes were averaged across all participants in Experiment 1. ***c***, Group average variability in hand positions over time as a function of visual uncertainty. Variability was computed as the area of the ellipse described by the SD of *x*- and *y*-positions. Each panel represents the change in area relative to the total average at that time point. The color-coding is identical to the one used in Figure 2 and the error bars indicate group averages ± SEM. * indicate pairwise comparisons with Bonferroni-corrected *p* < 0.005. In panels ***c***, lines on top of the panels indicate the pairwise comparison between conditions with low and infinite visual uncertainty in the slow (light gray line) and fast (dark gray line) conditions.

To quantify the effect of visual uncertainty and movement speed on feedback corrections at the group level, we computed the slopes for all participants at the five selected time points following the onset of the visual feedback presentation. Figure 3*a* shows the hand position at each of the five time points during the movement and stabilization phase averaged across trials without cursor shift. Naturally, these positions differed between the slow and fast condition due to the difference in movement speed. From 500 ms following the presentation of the visual feedback, the hand stabilized close to the target for both slow and fast conditions. Figure 3*b* demonstrates that 150 ms after the onset of the visual feedback, there was no observable influence of vision on the movement correction. However, starting at 300 ms, fast movements began to show negative slopes, signifying a modulation in lateral hand position in accordance with the shifted cursor. Even later during the movement, at 500 ms following the onset of vision, we observed negative slopes that scaled with visual uncertainty in both movement speed conditions, but the slopes were clearly larger during fast movements. Importantly, this difference between slow and fast conditions decreased again during even later time points (700 and 900 ms). We tested these effects using a repeated-measures ANOVA with visual uncertainty and movement speed as within participant factors which revealed a main effect of visual uncertainty starting at 300 ms after vision onset (300 ms: *F*_(3,45) _= 6.97, *p* = 0.0006, *η_p_*^2^ = 0.32; 500 ms: *F*_(3,45) _= 78.13, *p* < 10^−4^, *η_p_*^2^ = 0.84; 700 ms: *F*_(3,45) _= 140.95, *p* < 10^−4^, *η_p_*^2^ = 0.9; 900 ms: *F*_(3,45) _= 143.36, *p* < 10^−4^, *η_p_*^2^ = 0.91). In addition, slopes at 300 and 500 ms were significantly larger during fast compared with slow movements (300 ms: *F*_(1,15) _= 32.11, *p* < 10^−4^, *η_p_*^2^ = 0.68; 500 ms: *F*_(1,15) _= 49.73, *p* < 10^−4^, *η_p_*^2^ = 0.77). At 700 ms the relationship between fast and slow slopes showed a clear significant trend (*F*_(1,15) _= 10.01, *p* = 0.0064, *η_p_*^2^ = 0.4), but the effect was not significant anymore at 900 ms (*F*_(1,15) _= 2.41, *p* = 0.14). Taken together, these results highlight that feedback responses decreased with visual uncertainty. Additionally, there was a transitory period between 300 and 700 ms following the onset of vision during which the slopes were larger during fast movements.

10.1523/ENEURO.0262-24.2024.f3-1 Figure 3-1 **Lateral offset relative to the target at the end of the movement does not vary systematically with visual uncertainty or movement speed. (a)** Lateral (x-dimension) offset between the hand and the target midline at different timepoints following the onset of the visual feedback during trials without cursor shift in the slow movement condition. Lines in different shades of gray indicate the different force perturbation directions (black = rightward force, dark gray = no force, light gray = leftward force). The dashed horizontal line and the light gray shaded region mark the center and diameter of the target circle, respectively. **(b)** Same as (a) but for the fast movement condition. Download Figure 3-1, TIF file.

To assess whether participants exhibited a systematic offset relative to the center of the target, we analyzed the average lateral hand positions when no cursor shift was applied across the different visual uncertainties, movement speeds, and force perturbation directions. As expected, the lateral position of the hand throughout the movement depended on the direction of the applied force (Extended Data Fig. 3-1). Importantly, during the rightward force perturbations on which we focused our analysis, we observed no significant difference in the offset depending on the visual uncertainty at the final time point (900 ms: *F*_(3,45) _= 3.66, *p* = 0.0192; repeated-measures ANOVA). Secondly, while the offset was larger in the slow condition throughout the movement, there was no significant difference in the offset between slow and fast movements at the final time point (900 ms: *F*_(1,15) _= 5.14, *p* = 0.0386; repeated-measures ANOVA). Thus, while some participants exhibited an offset in their movement endpoint relative to the target, on average the hand landed in or close to the target at the end of the movement and endpoints did not systematically differ depending on the visual uncertainty or the movement speed.

To further investigate the influence of visual uncertainty on movement corrections, we tested whether movement variability also increased with visual uncertainty. Figure 3*c* shows the average area of the ellipse describing variability in *x*- and *y*-positions. For illustrative purposes, these variability values are shown as the difference to the average across all uncertainty conditions at each time point. We can observe that the variability in positions was larger during slow movements compared with fast up until 700 ms following the onset of visual feedback. The larger variability during slow movements might be linked to reduced temporal alignment of the traces, which is also visible in the wider distribution of the visual feedback onset times (Fig. 2*c*). Importantly, starting at 500 ms, we can see a positive relationship between the level of visual uncertainty and the variability (500 ms: *F*_(3,45) _= 15.19, *p* < 10^−4^, *η_p_*^2^ = 0.5; 700 ms: *F*_(3,45) _= 21.22, *p* < 10^−4^, *η_p_*^2^ = 0.59; 900 ms: *F*_(3,45) _= 21.71, *p* < 10^−4^, *η_p_*^2^ = 0.59; repeated-measures ANOVA). This finding provides additional support that the visual feedback contribution to the movement correction scaled with the signal reliability.

**Figure 4.**
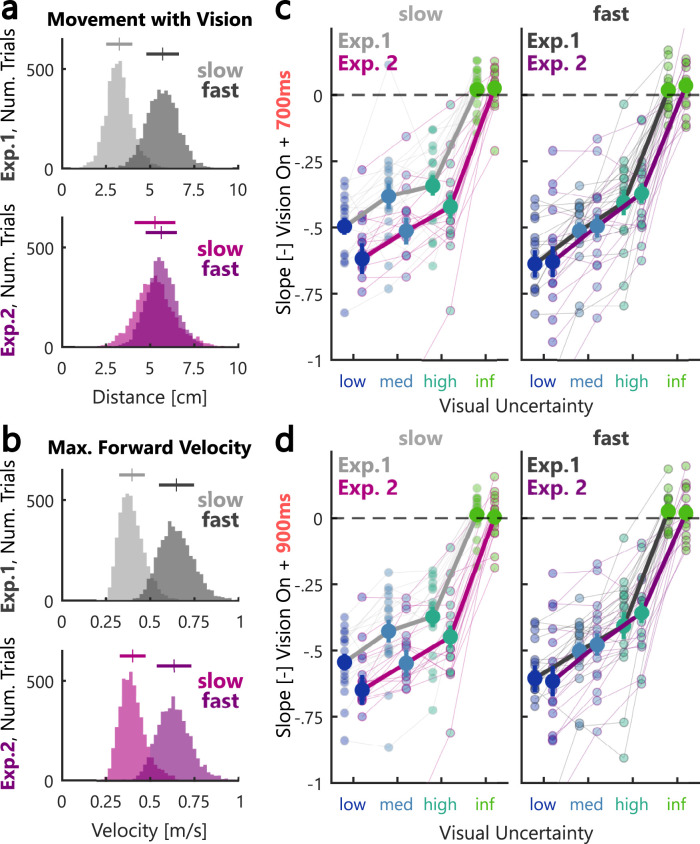
Longer viewing duration during slow movements increased visual feedback contribution. ***a***, Distribution of movement distances performed in the presence of visual feedback across all participants in Experiment 1 (top) and Experiment 2 (bottom). Slow movements are shown in light gray (Exp. 1) or pink (Exp. 2) and fast movements are shown in dark gray (Exp. 1) or purple (Exp. 2). Bars on the top of the histograms indicate mean values ± 1SD. ***b***, Distribution of maximum forward velocities across all participants in Experiment 1 (top) and Experiment 2 (bottom). Bars on the top of the histograms indicate mean values ± 1SD. ***c***, Slopes computed at 700 ms after the onset of the visual feedback as a function of visual uncertainty during slow (left) and fast (right) movements. Data from Experiment 1 is shown in gray and data of Experiment 2 in pink/purple. Thick lines indicate group averages and thin lines show individual participant data. ***d***, The same as ***c*** but at 900 ms after the onset of the visual feedback. The color-coding is identical to the one used in Figure 2 and the error bars indicate group averages ± SEM.

In Experiment 1 we presented the visual feedback for 100 ms during slow and fast movements. Consequently, visual information was available for a larger fraction of the hand path during fast movements. Hence, we performed a second experiment during which we matched the distance of the movement traveled with visual feedback between slow and fast movements. We used the average velocity to estimate the additional viewing time necessary to match the observable traveled distance and accordingly, increased the visual feedback presentation from 100 to 170 ms during slow movements in Experiment 2 (Fig. 4*a*, top and bottom panel; see Materials and Methods). Importantly, the average peak forward velocities remained identical between Experiments 1 and 2 [Fig. 4*b*, top and bottom panel; Exp. 1 (mean ± SD): slow: 0.4 m/s ± 0.07 m/s, fast: 0.65 m/s ± 0.1 m/s; Exp. 2: slow: 0.4 m/s ± 0.07 m/s, fast: 0.64 m/s ± 0.1 m/s]. At 500, 700, and 900 ms after vision onset, we observed slightly larger slopes during the slow movements in Experiment 2, while there was no such difference between experiments in the fast movements (Fig. 4*c*,*d*). Statistically, we observed an interaction between the effect of movement velocity and the experiment at 700 and 900 ms (Fig. 4*d*; 700 ms: *F*_(1,30) _= 6.37, *p* = 0.017, *η_p_*^2^ = 0.18; 900 ms: *F*_(1,30) _= 5.37, *p* = 0.027, *η_p_*^2^ = 0.15; repeated-measures ANOVA). In particular, while the slopes were significantly larger in the fast compared with the slow condition in Experiment 1, this difference was absent in Experiment 2. Hence, increasing the viewing duration of the visual feedback during slower movements increased the corrections for the cursor shift.

**Figure 5.**
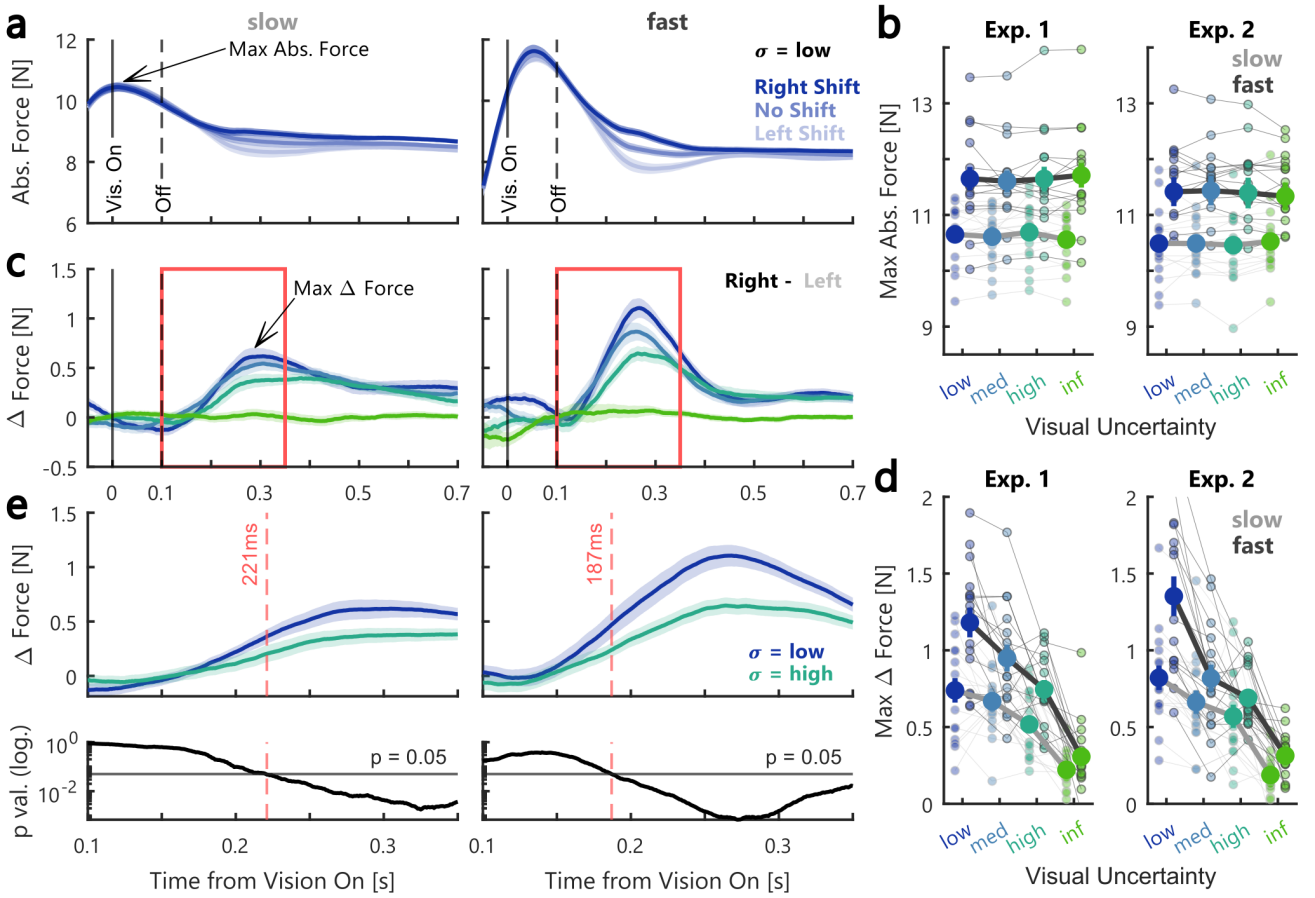
Lateral forces scaled with movement speed and visual uncertainty. ***a***, Group average absolute lateral force during rightward force perturbations and low visual uncertainty in the slow (left) and fast (right) movement condition in Experiment 1. Data was aligned to the onset time of the visual feedback. Shades of blue correspond to the three different cursor shift conditions for trials with low visual uncertainty. The shaded area corresponds to group mean ± SEM. ***b***, The maximum lateral force indicated in panel ***a*** as a function of visual uncertainty in Experiment 1 (left) and 2 (right). The dark gray lines represent fast and the light gray lines slow movements. Group averages are shown as thick lines and individual participants’ data as thin lines. ***c***, Difference in lateral forces to rightward–leftward cursor shift during slow (left) and fast (right) movements in Experiment 1. ***d***, The maximum delta force indicated in panel ***c*** as a function of visual uncertainty. ***e***, Top, Zoom-in of the delta lateral forces for conditions with low (blue) and high (turquoise) visual uncertainty during slow (left) and fast (right) movements. The area that is zoomed-in is depicted by a red box in panels ***c***. The shaded area corresponds to mean ± SEM. Dashed red lines mark the time point when the two traces started to diverge (determined by a running *t* test). Bottom, *p* values of running *t* tests over time. Dashed red lines mark the moment the *p* values fall below 0.05. The color-coding is identical to the one used in Figure 2 and the error bars indicate group averages ± SEM.

Previous studies have shown that control gains increase with the urgency to respond to a perturbation (Crevecoeur et al., 2013; Poscente et al., 2021). To assess whether this was the reason why we observed stronger responses to the visual feedback during fast movements, we compared the modulation of lateral interaction forces between participants’ hand and the robot handle during trials with a rightward perturbation load across movement speed conditions (Fig. 5*a*, data Exp. 1). Please note that as the lateral motion of the handle was not constrained during these trials, the recorded forces correspond to the lateral acceleration of the movement and, in absolute, do not reflect the exact forces participants applied on the handle. However, assuming constant mechanical impedance and given that the perturbation load magnitude was always the same, we can interpret the difference in measured forces as indicative of a modulation in participants’ feedback responses. Panels 5a show the absolute lateral forces averaged across trials with rightward (dark blue), no (medium blue), and leftward (light blue) cursor shifts and low visual uncertainty. It is visible that the lateral forces diverge with the direction of the cursor shift ca. 100 ms after the removal of the visual feedback. The maximum absolute lateral forces were clearly larger during fast compared with slow movements (Fig. 5*b*; Exp. 1: *F*_(1,15) _= 46.17, *p* < 10^−4^, *η_p_*^2^ = 0.75; Exp. 2: *F*_(1,15) _= 33.14, *p* < 10^−4^, *η_p_*^2^ = 0.69; repeated-measures ANOVA), which indicates an overall increase in responses to the force perturbation. We observed no influence of the cursor shift or the visual uncertainty on the peak lateral forces. To further investigate the influence of visual feedback uncertainty on the force responses, we computed the difference in forces during right and left cursor shifts (delta force; Fig. 5*c*). Next, we extracted the maximum delta force for each visual uncertainty condition (Fig. 5*d*). Using a repeated-measures ANOVA, we observed a main effect of movement velocity (Exp. 1: *F*_(1,15) _= 32.16, *p* < 10^−4^, *η_p_*^2^ = 0.68; Exp. 2: *F*_(1,15) _= 21.15, *p* = 0.0003, *η_p_*^2^ = 0.59) as well as a main effect of visual uncertainty (Exp. 1: *F*_(3,45) _= 42.81, *p* < 10^−4^, *η_p_*^2^ = 0.74; Exp. 2: *F*_(3,45) _= 48.8, *p* < 10^−4^, *η_p_*^2^ = 0.76) on the maximum delta force. Additionally, there was a significant interaction between movement velocity and visual uncertainty on the maximum delta force in Experiment 2 (Exp. 1: *F*_(3,45) _= 4.09, *p* = 0.01, *η_p_*^2^ = 0.21; Exp. 2: *F*_(3,45) _= 6.28, *p* = 0.0012, *η_p_*^2^ = 0.3). These findings demonstrate that the larger contribution of vision during fast movements observed in the slopes shown in Figure 3 could be linked to an increase in control gains with movement speed.

To determine the onset of the effect of visual uncertainty on the force differences, we computed running *t* tests to see when the force traces started to differ between the conditions with low and high visual uncertainty. We defined the onset as the first time step after the removal of the visual feedback at which the *p* value crossed below a threshold of 0.05. The estimated onset of the difference was 221 ms during slow movements and 187 ms during fast movements in Experiment 1. Further, we used bootstrapping with 10,000 iterations to estimate distributions of onset times for the slow and fast movements and observed no significant difference in onset times between movement speed conditions (data not shown). Thus, independent of movement speed, uncertainty-dependent responses to the visual feedback occurred at ∼200 ms following the onset of vision.

**Figure 6.**
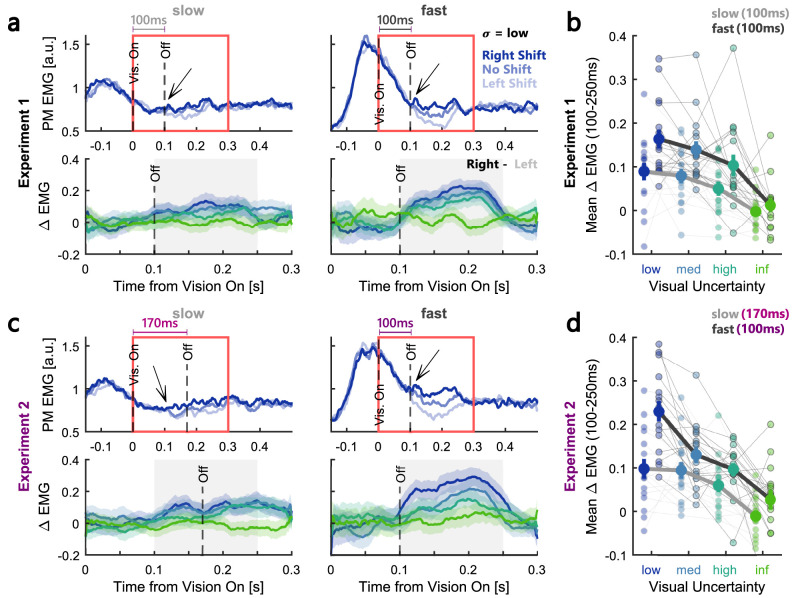
Agonist muscle responses scaled with movement speed and visual uncertainty. ***a***, Top, Group average pectoralis major EMG activity during rightward force perturbations and low visual uncertainty in the slow (left) and fast (right) movement condition in Experiment 1. Data was aligned to the onset time of the visual feedback. Shades of blue correspond to the three different cursor shift conditions. Dashed black lines indicate onset and offset of visual feedback. Bottom, Zoom-in of the difference in EMGs to rightward–leftward cursor shift during slow (left) and fast (right) movements in Experiment 1. The area that is zoomed-in is depicted by a red box in the top panels. In Experiment 1 the visual feedback duration was 100 ms for both slow and fast conditions as indicated by the gray/black time bars in the top panels. ***b***, EMG responses averaged across 100–250 ms following vision onset (gray-shaded area shown in ***a***, bottom panels) as a function of visual uncertainty. The slow condition is represented by light gray lines and the fast condition by dark gray lines. Group averages are shown as thick lines and individual participants’ data as thin lines. ***c***, Same as ***a*** but for Experiment 2. Note that in Experiment 2, the slow movement condition had a visual feedback duration of 170 ms while the fast movement condition was identical to Experiment 1 (100 ms feedback duration) as indicated by the pink/purple time bars in the top panels. ***d***, Same as ***b*** but for Experiment 2. The color-coding is identical to the one used in Figure 2 and the error bars indicate group averages ± SEM. See Extended Data Figure 6-1 for antagonist muscle (posterior deltoid) responses.

The increase in feedback responses with movement speed was also visible in the agonist muscle activity recorded in the pectoralis major muscle of the right shoulder [Fig. 6*a* (Exp. 1), *c* (Exp. 2)]. To investigate the influence of visual uncertainty on the modulation of muscle responses, we computed the average EMGs for each combination of cursor shift and visual uncertainty. The top panels of Figure 6*a*,*c* show the group average for each cursor shift during the condition with low visual uncertainty. For both slow and fast movements, we can see a separation of the traces depending on the cursor shift direction at ∼100 ms following the onset of the visual feedback. Next, to determine whether this effect was modulated by visual uncertainty, we computed the difference in EMGs between the right and left cursor shift for each visual uncertainty condition. The bottom panels in Figure 6*a*,*c* illustrate that this difference clearly decreased with increasing uncertainty. We then computed the average delta EMG across a time window ranging from 100 to 250 ms following the onset of the visual feedback [Fig. 6*b* (Exp. 1), *d* (Exp. 2)]. The average delta activity was overall larger during fast movements (Exp. 1: *F*_(1,15) _= 13.44, *p* = 0.0023, *η_p_*^2^ = 0.47; Exp. 2: *F*_(1,15) _= 44.58, *p* < 10^−4^, *η_p_*^2^ = 0.75; repeated-measures ANOVA) and showed a clear scaling with visual uncertainty (Exp. 1: *F*_(3,45) _= 15.38, *p* < 10^−4^, *η_p_*^2^ = 0.51; Exp. 2: *F*_(3,45) _= 24.84, *p* < 10^−4^, *η_p_*^2^ = 0.62; repeated-measures ANOVA). We observed no significant scaling with movement speed (Exp. 1: *F*_(1,15) _= 2.37, *p* = 0.14; Exp. 2: *F*_(1,15) _= 1.81, *p* = 0.2) or with visual feedback uncertainty (Exp. 1: *F*_(3,45) _= 1.86, *p* = 0.15; Exp. 2: *F*_(3,45) _= 1.36, *p* = 0.27) in the antagonist muscle activity (posterior deltoid; Extended Data Fig. 6-1), supporting that changes in movement speed and visual feedback uncertainty resulted in a modulation of visual feedback gains rather than muscle cocontraction.

10.1523/ENEURO.0262-24.2024.f6-1 Figure 6-1 **Antagonist muscle activity does not vary with visual feedback. (a)** Top: Group average posterior deltoid EMG activity during rightward force perturbations and low visual uncertainty in the slow (left) and fast (right) movement condition in experiment 1. Data was aligned to the onset time of the visual feedback. Shades of blue correspond to the three different cursor shift conditions. Dashed black lines indicate onset and offset of visual feedback. Bottom: Zoom-in of the difference in EMGs to rightward - leftward cursor shift during slow (left) and fast (right) movements in experiment 1. The area that is zoomed-in is depicted by a red box in the top panels. In experiment 1 the visual feedback duration was 100  ms for both slow and fast conditions as indicated by the gray/black time bars in the top panels. **(b)** EMG responses averaged across 100-250  ms following vision onset (gray-shaded area shown in (a) bottom panels) as a function of visual uncertainty. The slow condition is represented by light gray lines, the fast condition by dark gray lines. Group averages are shown as thick lines and individual participants’ data as thin lines. **(c)** Same as (a) but for experiment 2. Note that in experiment 2, the slow movement condition had a visual feedback duration of 170  ms while the fast movement condition was identical to experiment 1 (100  ms feedback duration) as indicated by the pink/purple time bars in the top panels. **(d)** Same as (b) but for experiment 2. The color-coding is identical to the one used in figure 2 and the error-bars indicate group-averages ± SEM. Download Figure 6-1, TIF file.

**Figure 7.**
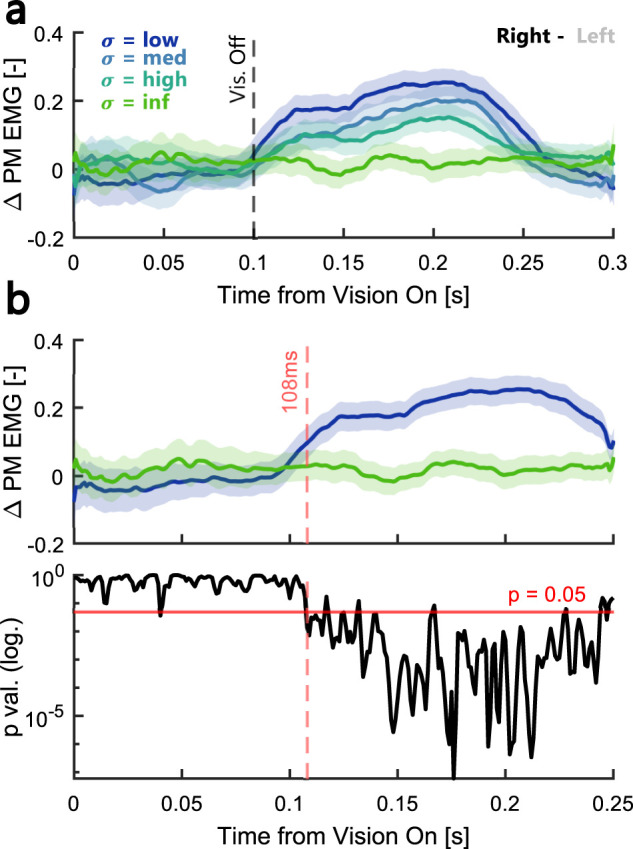
Latency of agonist muscle responses to visual feedback. ***a***, Difference in average EMG activity in the pectoralis major muscle to rightward–leftward cursor shifts averaged across all trials in the fast condition in Experiments 1 and 2. ***b***, Top, Zoom-in of the same delta EMGs for conditions with low (blue) and infinite (green) visual uncertainty. The dashed red line marks the time point when the two traces started to diverge (determined by a running *t* test). Bottom, *p* values of running *t* test over time. The dashed red line marks the moment the *p* value falls below 0.05. The color-coding is identical to the one used in Figure 2 and the shaded areas correspond to mean ± SEM.

Finally, we calculated the contrast in pectoralis major muscle activity between low and infinite visual uncertainty during fast movements averaged across both experiments to determine the latency of responses to the visual feedback. We observed a significant difference in muscle activity starting at 108 ms following the onset of vision (Fig. 7). Visual inspection of Figure 7 further shows that the intermediate visual uncertainty conditions start to diverge at a similar time even though significance is reached later due to smaller modulation amplitudes (∼150 ms following visual onset). These latencies are consistent with previous measures of rapid visual feedback responses to target or cursor jumps (Izawa and Shadmehr, 2008; Franklin et al., 2017; Cross et al., 2019).

**Figure 8.**
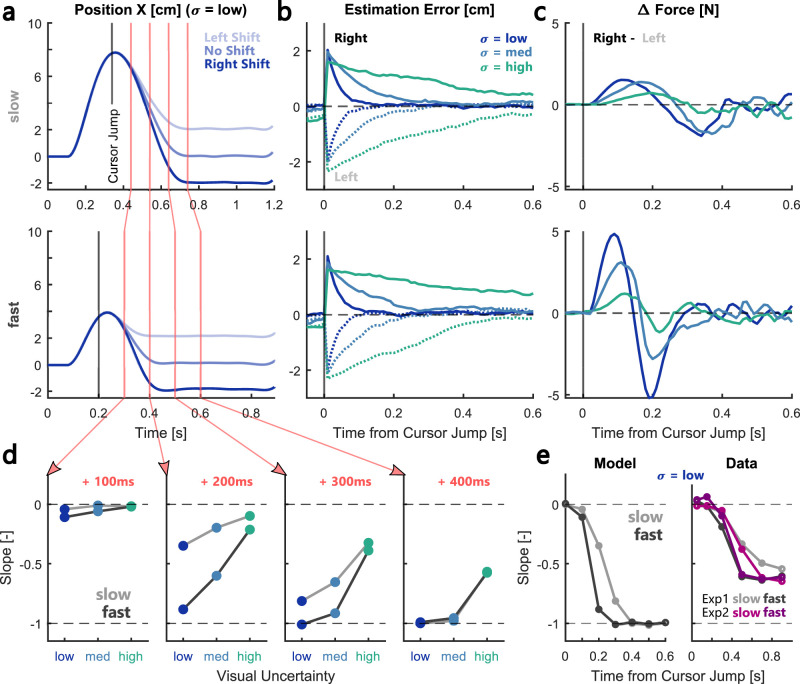
Model simulations reproduced effect of visual uncertainty and movement speed. ***a***, Simulated lateral positions over time during slow (top) and fast (bottom) movements with low visual uncertainty. The black line marks the moment the cursor jump was applied and the shades of blue correspond to the direction of the jump. Red vertical lines correspond to 100, 200, 300, and 400 ms after the cursor jump. ***b***, Average decay of the estimation error following the cursor jump during different visual uncertainties (blues and greens). The decay following a leftward cursor jump is shown as dotted lines, that following rightward jumps as full lines. ***c***, Difference in lateral forces to rightward–leftward cursor jumps during slow (top) and fast (bottom) simulated movements. ***d***, Slopes of the simulated movements at the four highlighted time points following the cursor jump as a function of visual uncertainty. Slow movements are shown as light gray lines and fast as dark gray lines. ***e***, The development of average slopes during low visual uncertainty over time from cursor jump/vision onset. Simulated data is shown on the left and experimental data on the right. Data of Experiment 1 is plotted in gray and data from Experiment 2 in pink/purple. See Extended Data Figures 8-1, 8-2 for comparison to simulations including signal-dependent sensory and motor noise. Refer to the following GitHub repository (https://github.com/annehoff/MultisensFBReaching) or Extended Data 1 for the model code used to generate this figure.

We implemented an LQG controller with a state estimator based on a Kalman filter to test whether such a continuous optimal integration model could capture the observed effects of visual uncertainty and movement speed on feedback corrections. As in our experiments, a 9 N constant load was applied to the simulated point mass as soon as it left the start position. For simplicity, contrary to the experiment, visual feedback was present during the entire length of the simulated movements as it is difficult to model a transient presentation of visual information in a linear system. Instead of the sudden flashing of the visual feedback, we introduced an instantaneous right- or leftward 2 cm cursor jump at the moment when the visual feedback was presented in the experiments. This cursor jump was not directly observable through the feedback equation (Eq. 4) and had to be estimated by the state estimator. In spite of all simplifying assumptions, the simulated trajectories closely resembled the observed behavior and displayed a similar divergence in lateral hand positions based on the direction of the cursor jump (Fig. 8*a*). Importantly, the model shows that the rate at which the state estimation error decreases following the cursor jump depends on visual uncertainty but does not differ between movement speeds (Fig. 8*b*). As shown in Figure 8*c*, the model accurately predicted an increase in the force modulation with visual feedback during fast movements, which was the result of a time-dependent increase in control gains.

10.1523/ENEURO.0262-24.2024.f8-1 Figure 8-1 **Model simulations with signal-dependent noise also reproduce effect of visual uncertainty and movement speed. (a)** Simulated lateral positions over time during slow (top) and fast (bottom) movements with low visual uncertainty. The black line marks the moment the cursor jump was applied and the shades of blue correspond to the direction of the jump. Red vertical lines correspond to 100, 200, 300, and 400  ms after the cursor jump. **(b)** Average decay of the estimation error following the cursor jump during different visual uncertainties (blues & greens). The decay following a leftward cursor jump is shown as dotted lines, that following rightward jumps as full lines. **(c)** Difference in lateral forces to rightward - leftward cursor jumps during slow (top) and fast (bottom) simulated movements. **(d)** Slopes of the simulated movements at the four highlighted timepoints following the cursor jump as a function of visual uncertainty. Slow movements are shown as light gray lines and fast as dark gray lines. **(e)** The development of average slopes during low visual uncertainty over time from cursor jump/vision onset. Simulated data is shown on the left and experimental data on the right. Data of experiment 1 is plotted in gray and data from experiment 2 in pink/purple. Refer to Extended data 1 for the model code used to generate this figure. Download Figure 8-1, TIF file.

10.1523/ENEURO.0262-24.2024.f8-2 Figure 8-2 **Comparison of Kalman and control gains between models without and with signal-dependent noise. (a)** Effect of visual sensory noise and movement speed on the norm of the block components of the Kalman gain matrix (Eq. 6) influencing the estimation of the lateral (x-dimension) position of the visual cursor. Data was aligned to the onset of the cursor jump in the simulations. Blue-green colors indicate different visual uncertainty levels. Dashed lines show values for slow movements and full lines those for fast movements. **(b)** Effect of visual sensory noise and movement speed on the control gains (Eq. 8) corresponding to the lateral offset between the simulated point mass and the cursor. The alignment and color-coding are the same as in (a). Refer to Extended data 1 for the model code used to generate this figure. Download Figure 8-2, TIF file.

Figure 8*d* depicts the temporal evolution of the simulated slopes at 100, 200, 300, and 400 ms following the cursor jump (Fig. 8*a*, red lines). The development of the slopes qualitatively resembles the results shown in Figure 3*b* and illustrates a similar effect of visual uncertainty and movement speed. Our model also allows us to isolate the relative contribution of visual uncertainty and movement speed on force modulations and slopes. To demonstrate this, we could assume an alternative scenario in which visual feedback contributes to the state estimate in a manner that is independent of sensory uncertainty. A simple way to simulate this in our model is to assume that the control command is computed based on the true state instead of the estimated state. In this “fully observable case” we assume that the system has complete knowledge of the state but the reasoning extends to situations in which we would assume that visual feedback is integrated based on a fixed weight independent of sensory uncertainty. In such a scenario, we no longer observe a scaling of the feedback responses with sensory uncertainty, and the estimation error is always zero (fully observable case) or decreases at the same rate independent of visual uncertainty (case with fixed weights). Instead, the effect of movement speed resulting from the change in control gains remains, explaining the difference in peak delta forces and the time-varying offset in estimated slopes between slow and fast movements.

Figure 8*e* depicts a direct comparison of model and data for slopes during the condition with low visual uncertainty. The dark and light gray lines show that it takes ∼300 ms following the cursor jump for the slope during the fast simulation to converge to the value of −1, whereas during the slow simulation it takes an additional 100 ms to reach the same value. This pattern looks similar when comparing the fast and slow movements in Experiments 1 and 2; however, there are some important differences between the model simulations and the data. Firstly, while the model starts to exhibit negative slopes at ∼100 ms following the visual perturbation, we only observed slopes significantly different from 0 at ca. 300 ms after vision onset in our experiments. This shift in time might be due to the fact that our model does not include time delays in the observed visual feedback. Secondly, the slopes we measured in our experiments converged to a value between −0.5 and −0.75, meaning that the cursor shift was not fully compensated. This divergence between the model and our data might have resulted from the fact that the visual feedback was only briefly presented in the experiments.

Importantly, for simplicity, our model only considers additive sensory and motor noise, and under this assumption, state estimation and control processes are independent from each other. However, it has long been suggested that the human sensorimotor system is subject to signal-dependent noise (Harris and Wolpert, 1998; Todorov, 2005), and in this case the independence of control and Kalman gains no longer holds. To investigate the impact of signal-dependent noise, we ran the same simulations using a model in which the motor noise and sensory noise scaled with the corresponding signals. The results of these simulations can be seen in Extended Data Figure 8-1. In general, we observed a similar behavior in terms of error decay, force modulations, and predicted slopes in the visual feedback responses. Further, the presence of signal-dependent noise did not influence the Kalman gains corresponding to the visual cursor positions (Extended Data Fig. 8-2). We only observed a small interaction between sensory uncertainty and movement speed on the estimated control gains corresponding to the cursor offset. Therefore, even if we assume that state estimation and control are not fully independent as in the case of signal-dependent noise, we still observed a dominant influence of movement speed on control gains and sensory uncertainty on state estimation.

In summary, our simulations show that the effect of visual uncertainty can be explained by an optimal state estimator that performs a continuous integration of sensory feedback and internal predictions, while the increase in movement speed induces a change in the control policy that maps state estimates onto motor commands. The results of our model mirror our empirical observations. Specifically, the finding that the contribution of visual feedback increased with viewing duration in Experiment 2 suggests that visual information was indeed accumulated and integrated continuously by the state estimator during the movement. Further, the speed-dependent modulation of forces and EMGs supports that the increased visual feedback response during fast movements was mitigated by an increase in control gains.

## Discussion

The present study aimed to investigate the influence of visual uncertainty on feedback corrections to combined visual and mechanical perturbations. To study the dynamics of the multisensory feedback responses, we varied both the movement time and the presentation duration of the visual feedback during the movement. Our results show that feedback corrections scaled with visual uncertainty and increased during faster movements. Further, we observed that extending the visual feedback duration during slow movements increased its contribution, leading to comparable levels of visual influence on feedback corrections during slow and fast conditions toward the movement end. We then leveraged a computational model to show in theory that there were two separable components underlying the observed behavior: the first is the integration of visual signals into the motor correction which depends on sensory uncertainty, and the second is the modulation of control gains with movement speed. Thus, we conclude that dynamic integration of vision and proprioception in our task was driven by a continuous accumulation of sensory evidence for state estimation. This estimate in turn interacted with the control policy which scaled with movement time.

Previous work has shown that humans behave close to optimal observers when combining perceptual priors and sensory evidence (Körding and Wolpert, 2004). Specifically, Izawa and Shadmehr (2008) showed that vigor of responses to target jumps depended on the change in relative uncertainty from the first to the second target location. They further demonstrated that the time course of these uncertainty-modulated responses could be predicted by a Kalman filter-based integration of internal priors and delayed sensory feedback, which led to a gradual convergence of the estimated target position toward the new target position. Our findings extend this model to multimodal perturbation responses by showing that feedback responses to force perturbations were modulated by visual uncertainty in a manner predicted by a Kalman filter.

In our second experiment, we observed that an extension of the visual feedback duration resulted in an increased contribution of vision to movement corrections. This observation supports the idea that visual evidence was integrated over the time course of the movement in a process resembling evidence accumulation described during decision-making. Specifically, drift-diffusion models predict that the rate at which the decision variable increases toward a decision threshold depends on the reliability of the sensory input (Ratcliff et al., 2016). In the context of reaching control, a stable Kalman filter predicts that estimation errors decay exponentially following a perturbation, and the rate of this decay is determined by the sensory feedback uncertainty. Further, by iteratively collecting noisy samples of the system state at each time point, a Kalman filter effectively implements a process resembling evidence accumulation over time. Hence, changing the duration of the visual feedback likely resulted in more information about the visual stimulus being accumulated by the state estimator and thus a larger reliance on vision. However, this assumption could not be directly tested in our model as it would require a change in the model structure to allow the addition and removal of visual signals which would in turn imply a recomputation of control and Kalman gains during the movement. Further, increasing the viewing duration necessarily resulted in a longer distance traveled with visual feedback, which makes it impossible to disentangle the influence of viewing duration and viewing distance in our experiments. Thus, to investigate the accumulation of sensory information during movement in more detail, future work may look more systematically at the influence of a larger variety of feedback presentation times on state estimation during movement.

While drift-diffusion models have been the standard account for evidence accumulation during decision-making processes, recent studies have proposed more advanced models to account for flexibilities in weighting sensory evidence as well as changes of mind during ongoing decision processes (Atiya et al., 2019; Prat-Ortega et al., 2021). For instance, Prat-Ortega et al. (2021) demonstrated that point-attractor dynamics can explain transitions between primary and recency effects of evidence weighting depending on the stimulus uncertainty or duration while drift-diffusion models can account for these observations only by changing model parameters, such as setting absorbing or reflecting decision bounds. However, these more complicated dynamics of decision-making likely come to play at stimulus durations exceeding the 100–170 ms used in our experiments. Thus, future work exploring longer feedback durations may also consider more complex evidence accumulation dynamics and investigate whether they are consistent with tracking of sensory evidence using a Kalman filter.

As an alternative to evidence accumulation accounts of decision-making, it has been suggested that decisions are instead reached using an urgency gating process. Contrary to the drift-diffusion model, urgency gating does not assume that sensory evidence is accumulated over time and has been able to explain the influence of transient increases in sensory evidence applied at different times during the decision-making process (Carland et al., 2016). Instead, this model proposes that sensory evidence is passed through a low-pass filter with a short time constant and then multiplied with an urgency signal that grows over time (Cisek et al., 2009). Interestingly, a growing urgency signal effectively acts like a gain applied to the current sensory evidence, which closely resembles the influence of movement speed observed in our results. According to Optimal Feedback Control, motor commands are computed by mapping control gains onto the estimated state of the system. An increase in movement speed leads to a rise in urgency to respond to the perturbation which has been shown to result in an increase in control gains (Crevecoeur et al., 2013; Dimitriou et al., 2013; Poscente et al., 2021). In our experiments this resulted in a transient phase during which the visual compensation appeared larger during fast movements, while the actual estimation error was not influenced by movement speed but was simply mapped onto a different control function. Since we manipulated response urgency by setting different timing constrains on the movement, we cannot disentangle the influence of urgency or movement speed on feedback responses in our experiments. However, a recent study by Česonis and Franklin (2020) demonstrated that the intensity of visuomotor feedback gains scaled non-monotonically with the time interval between perturbation onset and arrival at the target rather than movement speed at perturbation onset, suggesting that “time-to-target” is a valid measure of response urgency. Our manipulation of movement time also influenced the time between perturbation onset and reaching the target, and thus our experiments address a similar definition of response urgency.

Importantly, urgency gating models of decision-making assume that only novel evidence should influence the decision process which is implemented by assuming a leaky integration process. In our study the hand was moving during the presentation window of the visual feedback; hence, each moment in time presented novel evidence about the hand location which was integrated by the state estimator. While resolving the debate between evidence-accumulation and urgency-gating accounts of decision-making is beyond the scope of our study, we demonstrate here that multisensory feedback corrections are influenced by both of these processes. In particular, we suggest that evidence accumulation is performed by the state estimator whereas urgency signals modulate the control policy with which the sensorimotor system responds to incoming feedback signals. Thus, these two processes can be well separated within the context of reaching control.

In theory, a complete separability of state estimation and control processes only exists in the presence of purely additive noise signals. However, it is known that the sensorimotor system is subject to signal-dependent noise (Harris and Wolpert, 1998; Todorov, 2005). In particular, it has been shown that motor noise increases with the average applied force (Jones et al., 2002). To capture this property of sensorimotor systems, we demonstrated that even in the presence of signal-dependent noise, movement time predominantly influenced control gains while sensory uncertainty determined Kalman gains resulting in very similar predicted behavior. Besides the fact that signal-dependent noise requires specific mathematical treatment, the approximation that separable processes underlie estimation and control is in line with current theories of decision-making, which suggest that sensory evidence and urgency signals are computed by prefrontal cortex and basal ganglia, respectively, and converge in motor cortex areas (Thura et al., 2022). It remains for future work to investigate whether similar brain networks also underlie online movement control.

Our results provide additional support that decision-related evidence accumulation and movement execution co-occur in time and that decision variables are continually transferred to motor areas during movement as has been suggested by previous work. For example, Selen et al. (2012) demonstrated that the accumulated sensory evidence during a decision process was continuously transferred to the motor system during movement preparation by showing that long-latency reflex gains scaled with the accumulated evidence. Further, the occurrence of changes of mind after the onset of a movement demonstrates that the decision process overlaps with movement execution (Resulaj et al., 2009; Visser et al., 2023). Other studies have shown that speed constraints imposed on decision-making can influence the speed of subsequent movements and vice versa (Carsten et al., 2023). In line with such bidirectional influences between decision-making and movement control, Kelly and O’Connell (2013) showed that a centroparietal positivity component in an EEG study scaled both with coherence of a random dot motion stimulus as well as with the reaction time of the subsequent decision. The authors linked this effect to a reduction in alpha-band power preceding faster responses which is commonly interpreted as an increase in attention to the stimulus. Although speculative, a similar process might underlie the increase in response to visual feedback during faster movements we observed in our data.

The existence of varying processing delays within different sensory modalities has inspired the proposal that initial movement corrections might rely on intramodal estimates until multimodal state estimates become available later on during processing (Oostwoud Wijdenes and Medendorp, 2017). A recent study has shown that initial responses to visual and proprioceptive perturbations exhibited additive effects whereas interactions between these feedback modalities only became visible ∼100 ms after the onset of responses to visual feedback (Keyser et al., 2023). While the authors interpreted this as evidence against a Kalman filter-based integration, we demonstrated here that a linear state estimator indeed predicts such additive contributions of proprioceptive and visual errors to feedback responses. Nonetheless, a Kalman filter is a description of the behavioral output and does not make assumptions of how the brain produces this behavior. Previous work has shown that different pathways exist both within the proprioceptive and visual modalities (Pruszynski et al., 2010; Scott, 2016; Cross et al., 2019), such that the resulting neural command sent to the muscles is a combination of both independent and combined pathways. The question is where in the brain information from these different pathways is combined for multimodal processing. For example, Bakola et al. (2010) used fluorescent tracers in cynomolgus monkeys to show that neuronal populations in parietal cortex receive both visual and proprioceptive inputs specifically linked to limb positioning in space. Further, a recent study has shown that limb afferent feedback and visual information about limb and target location converge on similar neuron populations in primary motor cortex of monkeys (Cross et al., 2024). Given the latencies that we observed for the visual feedback in our study (ca. 100 ms), these responses likely relied both on separate and combined neural pathways. Hence, an important question for neurophysiological studies is to investigate how multimodal pathways complement separate sensory processing to produce a behavioral output that matches the prediction of a Kalman filter.

In summary, our results show that multimodal feedback responses during movement not only depend on sensory uncertainty but are further influenced by movement speed and visual feedback duration. Importantly these two influences can be respectively linked to the urgency as a feature of the control policy and to evidence accumulation over time as a property of the state estimator. From this perspective, these two components of behavior observed across decision-making and motor control tasks can be dissociated and attributed to well-defined computational operations of the sensorimotor system.

## Synthesis

Reviewing Editor: David Franklin, Technische Universitat Munchen Decisions are customarily a result of the Reviewing Editor and the peer reviewers coming together and discussing their recommendations until a consensus is reached. When revisions are invited, a fact-based synthesis statement explaining their decision and outlining what is needed to prepare a revision will be listed below. The following reviewer(s) agreed to reveal their identity: Luc Selen. Note: If this manuscript was transferred from JNeurosci and a decision was made to accept the manuscript without peer review, a brief statement to this effect will instead be what is listed below.

Your manuscript has been re-reviewed by the two original reviewers. They both appreciate the revisions and clarifications that you have made to the manuscript. They also highlight the very usable code to run the simulations. However, after discussion we believe that are still a few issues that need to be resolved before we can accept the manuscript. In particular we all have concerns about the force measurements that are included in the manuscript as the handle endpoint and therefore the force transducer is free to move in the lateral axis (no channel trials) meaning that the measured forces are not actually the force produced by the participant. This is because the force produced will affect the acceleration, only part of which is picked up by the force transducer. Instead, on freely moving trials you are likely better off either plotting the acceleration or velocity of movement (e.g. I use this in several of my own papers). Or perhaps we are missing information about the constraints on these specific trials to resist motion in this axis? In addition to this issue, there are several further points that need to be addressed in a revision. Please consider the further comments of the reviewers as you revise the manuscript, and provide a point-by-point response explaining your revisions.

Specific comments.

Reviewer 1:

I want to thank the authors for taking my comments seriously. They have addressed most of my technical concerns. Below are a few conceptual concerns that might need more consideration.

1. [Major point 1.a] I understand that authors manipulated urgency, but in different urgency conditions, effectively participants differ in their movement speeds. How do you dissociate the effect of "urgency" from "movement speed" as these were correlated? In other words, the authors interpret their results based on urgency, but it's unclear whether the effects are due to urgency or movement speed (e.g., Figure 5 or 6).

I think reviewer #1 had the same concern about equating urgency and speed. I don't think the authors response fully addressed (reviewer 1) #2, #7 or (reviewer 2) #1.a. The authors clarified the validity of their manipulation of "urgency" conditions in terms of task design, but did not explicitly discuss the implications of this confound on their result (I understand in the model urgency and speed can be separated, what about in observed behavior?).

2. [Major point 1.b] Thank the authors for clarifying their modeling of evidence accumulation via a Kalman filter model. What the authors have done in terms of modeling is good enough for me, I just want to raise one conceptual point:

The authors stated in line 97-98:

"The rate of this convergence depends on the sensory uncertainty in a similar way as the drift rate of sensory evidence accumulation during perceptual decision making (Ratcliff, 1978)"

Also, in the authors' response, they said "Our results demonstrate that the rate of this integration process is influenced by the uncertainty of the visual feedback in a similar way as the diffusion [I assume authors meant drift rate] rate in a decision processed is influenced by the coherence of a sensory stimulus."

I want to highlight that recent research has challenged the view that uncertainty in (perceptual and other type of) decisions is entirely a drift-diffusion process (which is the standard model in the field for a long time and the assumption taken by the authors).

In recent literature, when alternative mechanisms are considered (Prat-Ortega et al., 2021, Wang et al., 2023), "uncertainty" or "coherence" are shown to be better characterized by other types of models (e.g., point attractor model) as opposed to evidence accumulation (which assumes a line attractor). By assuming a drift-diffusion alone process, these alternative mechanisms like point-attractor dynamics will be captured by the drift term (the residual diffusion noise term will be affected as well). The authors could discuss limitations of the interpretation of drift rate (or the limitation of using a pure evidence accumulation model), as drift rate can reflect other processes (Atiya et al., 2019, Prat-Ortega et al., 2021, Wang et al., 2023) that are unmodelled and does not necessarily reflect a true drift-diffusion process.

The effect of visual feedback uncertainty in the current paper is also likely a combination of a drift-diffusion process, and some other network attractor dynamics that govern motor response. It's a minor point, but just want to point out that the link between visual uncertainty and response slope is likely not simply due to a drift-diffusion process.

References:

Prat-Ortega, G., Wimmer, K., Roxin, A. et al. Flexible categorization in perceptual decision making. Nat Commun 12, 1283 (2021). https://doi.org/10.1038/s41467-021-21501-z

Wang, S., Falcone, R., Richmond, B. et al. Attractor dynamics reflect decision confidence in macaque prefrontal cortex. Nat Neurosci 26, 1970-1980 (2023). https://doi.org/10.1038/s41593-023-01445-x

Atiya, N.A., Rañó, I., Prasad, G. et al. A neural circuit model of decision uncertainty and change-of-mind. Nat Commun 10, 2287 (2019). https://doi.org/10.1038/s41467-019-10316-8

Reviewer 2:

The authors have greatly improved the manuscript and provided extensive answers to the earlier raised questions and concerns. The only remaining issue is the measurement and analysis of forces. A force transducer only provides force if it is bolted down (or nearly bolted down). Since you have no channels, you are effectively measuring acceleration. Either rewrite everything with the forces, or remove it altogether. I don't think it is a necessary ingredient for the story. Furthermore, I found one of you figures confusing (see below). For the rest, great paper.

Abstract now reads nicely, changes greatly improved it.

Ln 227-230: Sure, you have a force transducer, but this can only measure forces if it is stationary (force and acceleration are non-dissociable!).

Ln 252: Forces you cannot compute if you do not introduce a channel. Essentially you are looking at acceleration.

Ln 103: You miss a full stop after the reference.

Ln 155: was it a 2D cloud? Or only along the y-axis?

I am lost in Figure 6. First, panel D is about short vs long viewing duration, right? Not slow vs fast movement as the legend says. Furthermore, from the text I understood that longer viewing durations result in stronger responses (i.e. an accumulation effect), but in this figure (panels C and D) I see the opposite! Where do I make an interpretation mistake?

## Author Response

Answers to Reviewers We thank the reviewers for taking the time to read our previous revisions and we are happy that many of the earlier concerns could be resolved. Below we provide our answers to the remaining comments of the reviewers.

Please note that the indicated page and line references correspond to the revised version of our manuscript. Page and line references that refer to parts of the manuscript that were added or changed are highlighted in red, whereas text references to sections that were already contained in the original manuscript are printed in blue font. Similarly, text passages that we changed or added to the original manuscript are highlighted in red in our answers and in the text of the revised manuscript.

Reviewer #1:

I want to thank the authors for taking my comments seriously. They have addressed most of my technical concerns. Below are a few conceptual concerns that might need more consideration.

1. [Major point 1.a] I understand that authors manipulated urgency, but in different urgency conditions, effectively participants differ in their movement speeds. How do you dissociate the effect of "urgency" from "movement speed" as these were correlated? In other words, the authors interpret their results based on urgency, but it's unclear whether the effects are due to urgency or movement speed (e.g., Figure 5 or 6).

I think reviewer #1 had the same concern about equating urgency and speed. I don't think the authors response fully addressed (reviewer 1) #2, #7 or (reviewer 2) #1.a. The authors clarified the validity of their manipulation of "urgency" conditions in terms of task design, but did not explicitly discuss the implications of this confound on their result (I understand in the model urgency and speed can be separated, what about in observed behavior?).

Due to our manipulation of time constrains, the influence of response urgency and movement speed cannot be distinguished in our experiments. To do this it would be necessary to manipulate urgency without influencing movement speed.

In general, it is not easy to implement an urgency manipulation which does not influence movement kinematics. For example, studies on urgent decision making have manipulated urgency by varying the time between trials to encourage participants to make faster decisions to obtain more rewards in a shorter amount of time (i.e. Cisek et al. 2009). However, applied to movements such a design would likely also result in different movement speeds. Alternatively, we could have applied the visual cursor shift at different distances relative to the target. In such a design the urgency to respond to the perturbation would increase if it is applied closer to the target. However, even in this design the forward velocity at perturbation onset would differ depending on when during the movement the perturbation is applied. To distinguish "time to target" and differences in movement kinematics, a recent study by Česonis &Franklin (2020) trained participants to follow specific velocity profiles to vary the movement time for the same reaching distance. They observed that feedback intensity scaled with the time between perturbation onset and reaching the target and not with the speed of the movement at perturbation onset. Consequently, they defined the "time to target" as a direct measure of response urgency which determines feedback gains. Although less fine grained, our manipulation of movement time effectively also changed the time between perturbation onset and reaching the target. Thus, while we do acknowledge that there exist other ways to manipulate response urgency during movements, we believe that imposing different timing constraints on a movement is a valid and intuitive way to manipulate the urgency to respond to a perturbation.

To address the confound between response urgency and movement speed as well as alternative ways to manipulate urgency, we added the following to the discussion of our revised manuscript (p. 29/30, lines 666-679): "According to Optimal Feedback Control, motor commands are computed by mapping control gains onto the estimated state of the system. An increase in movement speed leads to a rise in urgency to respond to the perturbation which has been shown to result in an increase in control gains (Crevecoeur et al., 2013; Dimitriou et al., 2013; Poscente et al., 2021). In our experiments this resulted in a transient phase during which the visual compensation appeared larger during fast movements, while the actual estimation error was not influenced by movement speed but was simply mapped onto a different control function. Since we manipulated response urgency by setting different timing constrains on the movement, we cannot disentangle the influence of urgency or movement speed on feedback responses in our experiments. However, a recent study by Česonis and Franklin (2020) demonstrated that the intensity of visuomotor feedback gains scaled non-monotonically with the time interval between perturbation onset and arrival at the target rather than movement speed at perturbation onset, suggesting that "time-to-target" is a valid measure of response urgency. Our manipulation of movement time also influenced the time between perturbation onset and reaching the target and thus our experiments address a similar definition of response urgency." References:

Cisek, P., Puskas, G. A., &El-Murr, S. (2009). Decisions in changing conditions: The urgency-gating model. Journal of Neuroscience, 29(37), 11560-11571. Česonis, J., &Franklin, D. W. (2020). Time-to-Target Simplifies Optimal Control of Visuomotor Feedback Responses. ENeuro, 7(2).

2. [Major point 1.b] Thank the authors for clarifying their modeling of evidence accumulation via a Kalman filter model. What the authors have done in terms of modeling is good enough for me, I just want to raise one conceptual point:

The authors stated in line 97-98: "The rate of this convergence depends on the sensory uncertainty in a similar way as the drift rate of sensory evidence accumulation during perceptual decision making (Ratcliff, 1978)" Also, in the authors' response, they said "Our results demonstrate that the rate of this integration process is influenced by the uncertainty of the visual feedback in a similar way as the diffusion [I assume authors meant drift rate] rate in a decision processed is influenced by the coherence of a sensory stimulus." I want to highlight that recent research has challenged the view that uncertainty in (perceptual and other type of) decisions is entirely a drift-diffusion process (which is the standard model in the field for a long time and the assumption taken by the authors).

In recent literature, when alternative mechanisms are considered (Prat-Ortega et al., 2021, Wang et al., 2023), "uncertainty" or "coherence" are shown to be better characterized by other types of models (e.g., point attractor model) as opposed to evidence accumulation (which assumes a line attractor). By assuming a drift-diffusion alone process, these alternative mechanisms like point-attractor dynamics will be captured by the drift term (the residual diffusion noise term will be affected as well). The authors could discuss limitations of the interpretation of drift rate (or the limitation of using a pure evidence accumulation model), as drift rate can reflect other processes (Atiya et al., 2019, Prat-Ortega et al., 2021, Wang et al., 2023) that are unmodelled and does not necessarily reflect a true drift-diffusion process.

The effect of visual feedback uncertainty in the current paper is also likely a combination of a drift-diffusion process, and some other network attractor dynamics that govern motor response. It's a minor point, but just want to point out that the link between visual uncertainty and response slope is likely not simply due to a drift-diffusion process.

References:

Prat-Ortega, G., Wimmer, K., Roxin, A. et al. Flexible categorization in perceptual decision making. Nat Commun 12, 1283 (2021). https://doi.org/10.1038/s41467-021-21501-z Wang, S., Falcone, R., Richmond, B. et al. Attractor dynamics reflect decision confidence in macaque prefrontal cortex. Nat Neurosci 26, 1970-1980 (2023). https://doi.org/10.1038/s41593-023-01445-x Atiya, N.A., Rañó, I., Prasad, G. et al. A neural circuit model of decision uncertainty and change-of-mind. Nat Commun 10, 2287 (2019). https://doi.org/10.1038/s41467-019-10316-8 Thank you for referring us to these references. We acknowledge that we are assuming a rather simple process of evidence accumulation by using a linear Kalman filter. We believe that accounting for more complex point attractor dynamics in sensory evidence accumulation during movement is a very interesting avenue for future research, especially when considering longer feedback durations as primacy/recency effects and changes-of-mind are more likely to play a role at longer exposure times to sensory stimuli.

We made the following additions to the discussion of our revised manuscript (p.28/29, lines 637-657) to integrate these ideas: "Hence, changing the duration of the visual feedback likely resulted in more information about the visual stimulus being accumulated by the state estimator, and thus a larger reliance on vision. However, this assumption could not be directly tested in our model as it would require a change in the model structure to allow the addition and removal of visual signals which would in turn imply a re-computation of control and Kalman gains during the movement. Further, increasing the viewing duration necessarily resulted in a longer distance travelled with visual feedback, which makes it impossible to disentangle the influence of viewing duration and viewing distance in our experiments. Thus, to investigate the accumulation of sensory information during movement in more detail, future work may look more systematically at the influence of a larger variety of feedback presentation times on state estimation during movement.

While drift-diffusion models have been the standard account for evidence accumulation during decision-making processes, recent studies have proposed more advanced models to account for flexibilities in weighting sensory evidence as well as changes of mind during ongoing decision processes (Atiya et al., 2019; Prat-Ortega et al., 2021). For instance, Prat-Ortega et al. (2021) demonstrated that point-attractor dynamics can explain transitions between primary and recency effects of evidence weighting depending on the stimulus uncertainty or duration while drift-diffusion models can account for these observations only by changing model parameters, such as setting absorbing or reflecting decision bounds. However, these more complicated dynamics of decision-making likely come to play at stimulus durations exceeding the 100-170ms used in our experiments. Thus, future work exploring longer feedback durations may also consider more complex evidence accumulation dynamics and investigate whether they are consistent with tracking of sensory evidence using a Kalman filter. " Reviewer #2:

The authors have greatly improved the manuscript and provided extensive answers to the earlier raised questions and concerns. The only remaining issue is the measurement and analysis of forces. A force transducer only provides force if it is bolted down (or nearly bolted down). Since you have no channels, you are effectively measuring acceleration. Either rewrite everything with the forces, or remove it altogether. I don't think it is a necessary ingredient for the story. Furthermore, I found one of you figures confusing (see below). For the rest, great paper.

Abstract now reads nicely, changes greatly improved it.

Thank you for this comment.

Ln 227-230: Sure, you have a force transducer, but this can only measure forces if it is stationary (force and acceleration are non-dissociable!).

We agree that the forces we report correspond to the acceleration of the moving handle and we clarified this in the method section (p. 10, lines 224-228): "Forces applied to the handle of the KINARM robot were recorded using an ATI force sensor located at the top of the handle and mapped into the x- and y-coordinate system of the experimental workspace using the Dexterit-E software. As the lateral motion of the handle was not constraint during the movement, these recorded forces correlate with the acceleration of the movement." ... and the result section (p. 22, lines 480-486) of our revised manuscript: "Previous studies have shown that control gains increase with the urgency to respond to a perturbation (Crevecoeur et al., 2013; Poscente et al., 2021). To assess whether this was the reason why we observed stronger responses to the visual feedback during fast movements, we compared the modulation of lateral interaction forces between participants' hand and the robot handle during trials with a rightward perturbation load across movement speed conditions (Fig. 5a, data Exp. 1). Please note that as the lateral motion of the handle was not constraint during these trials, the recorded forces correlate with the lateral acceleration of the movement." Ln 252: Forces you cannot compute if you do not introduce a channel. Essentially you are looking at acceleration.

We believe that force signals are meaningful even without using force channels. In fact, the acceleration signals obtained from numerical differentiation were less sensitive to the difference in visual uncertainty due to filtering, and the acceleration signals encoded by the accelerometers in the handle were very noisy and also required additional filtering (see added figure below). Thus, the encoded forces, which require neither filtering nor numerical derivation, gave us the most reliable signal and allowed us to establish a direct link between the behavior and the recorded EMG responses. For these reasons, we believe that our force data are reliable and meaningful and we have a clear preference for keeping them while being fully aware that they should not be interpreted as the voluntary forces produced against a channel wall. However, we are willing to replace them with the acceleration signals from numerical differentiation if the Reviewer and Editor prefer this change.

Below we added a figure comparing derived lateral velocity, derived lateral acceleration, encoded lateral acceleration, and encoded lateral force to demonstrate the differences in noise level and temporal sensitivity in these signals. The traces display the average {plus minus} SEM across participants in the low (blue) and high (turquoise) visual uncertainty conditions. This contrast was used to estimate the onset of the modulation in feedback responses depending on the degree of visual uncertainty. Please observe that estimates based on velocity were late, and estimates based on acceleration (derived or encoded) were noisier. Taking this into account, we opted for the encoded force (knowing it is proportional to acceleration) as it gave us the least noisy and most reliable onset estimate.

Figure. Comparison of onsets in response modulation with visual uncertainty obtained with lateral velocity, acceleration, and force data. (a) Delta lateral velocity derived from filtered position data (rightward-leftward cursor shift) for conditions with low (blue) and high (turquoise) visual uncertainty during slow movements. The estimated onset of the visual feedback response modulation with uncertainty is marked by the dashed vertical line. (b) Same as (a) but for the lateral acceleration derived from the filtered velocity signal. (c) Same as (a) but for the acceleration encoded by the accelerometers in the robot handle. The encoded acceleration was filtered using a low-pass, fourth-order, dual-pass Butterworth filter with a cutoff frequency of 50Hz before computing the average delta traces shown here. (d) Same as (a) but for the lateral force encoded by the force sensors in the robot handle. This is identical to the data reported in Fig. 5e. (e) P-values of running t-tests over time. Dashed colored vertical lines mark the moment the p-values fall below 0.05 for the corresponding signal.

Ln 103: You miss a full stop after the reference.

Thank you for pointing this out. We added the missing full stop on page 5, line 101 of the revised manuscript: "On the one hand, the control gains, with which the system responds to sensory errors, are known to be tuned to both the dynamics of the environment (Maurus et al., 2023) and the urgency to respond to a perturbation (Crevecoeur et al., 2013; Dimitriou et al., 2013; Oostwoud Wijdenes et al., 2011; Poscente et al., 2021). On the other hand, considering additive noise as a first approximation, the dynamics of state-estimation only depend on the statistics of the noise disturbances, and not on the urgency." Ln 155: was it a 2D cloud? Or only along the y-axis? We used 2-dimensional cursor clouds with equal standard deviation in x- and y-dimensions in our experiments. We clarified this in the method section of the revised manuscript (p. 7, lines 152-154): "The cursor clouds were composed of 25 dots of 0.2cm radius. The position of each cursor was drawn randomly from a normal distribution centered on the hand position with a standard deviation of 1cm (medium uncertainty) or 2cm (high uncertainty) in x- and y-dimensions." I am lost in Figure 6. First, panel D is about short vs long viewing duration, right? Not slow vs fast movement as the legend says. Furthermore, from the text I understood that longer viewing durations result in stronger responses (i.e. an accumulation effect), but in this figure (panels C and D) I see the opposite! Where do I make an interpretation mistake? Figure 6 panel d compares the delta muscle activity averaged over a time window from 100-250ms after vision onset between the slow (in gray) and the fast (in black) movement condition in experiment 2. The slow movement condition in experiment 2 has the longer viewing duration (170ms instead of 100ms, indicated by dashed black lines in Fig. 6c, left panels), while the fast movement condition was identical to experiment 1 (100ms viewing duration in both cases). Clearly the EMG responses were stronger in the fast movement condition also in experiment 2 (C, panels on the right), which makes sense given the increased feedback gains during faster movements. The increased response to the longer viewing duration when comparing the slopes in the slow conditions across experiments is not directly visible from the EMG traces. This is because the muscle activity captures the very early response to the visual feedback and differences due to viewing duration where visible in the slopes only at the very end of the movement.

We added the viewing duration to the figure and legend to clarify the differences between the two experiments (p. 40/41, lines 929-944):

Figure 6. Agonist muscle responses scaled with movement speed and visual uncertainty. (a) Top: Group average pectoralis major EMG activity during rightward force perturbations and low visual uncertainty in the slow (left) and fast (right) movement condition in experiment 1. Data was aligned to the onset time of the visual feedback. Shades of blue correspond to the three different cursor shift conditions. Dashed black lines indicate onset and offset of visual feedback. Bottom: Zoom-in of the difference in EMGs to rightward - leftward cursor shift during slow (left) and fast (right) movements in experiment 1. The area that is zoomed-in is depicted by a red box in the top panels. In experiment 1 the visual feedback duration was 100ms for both slow and fast conditions as indicated by the gray/black time bars in the top panels. (b) EMG responses averaged across 100-250ms following vision onset (gray-shaded area shown in (a) bottom panels) as a function of visual uncertainty. The slow condition is represented by light gray lines, the fast condition by dark gray lines. Group averages are shown as thick lines and individual participants' data as thin lines. (c) Same as (a) but for experiment 2. Note that in experiment 2, the slow movement condition had a visual feedback duration of 170ms while the fast movement condition was identical to experiment 1 (100ms feedback duration) as indicated by the pink/purple time bars in the top panels. (d) Same as (b) but for experiment 2. The color-coding is identical to the one used in figure 2 and the error-bars indicate group-averages {plus minus} SEM. See supporting figure 6-1 for antagonist muscle (posterior deltoid) response.

For consistency, we made the same changes to supporting figure 6-1 (p. 42, lines 974-989).

## References

[B1] Alais D, Burr D (2004) The ventriloquist effect results from near-optimal bimodal integration. Curr Biol 14:257–262. 10.1016/j.cub.2004.01.029 14761661

[B2] Åström KJ (1970) *Introduction to stochastic control theory*. New York: Academic Press.

[B3] Atiya NAA, Rañó I, Prasad G, Wong-Lin KF (2019) A neural circuit model of decision uncertainty and change-of-mind. Nat Commun 10:2287. 10.1038/s41467-018-07882-8 31123260 PMC6533317

[B4] Bakola S, Gamberini M, Passarelli L, Fattori P, Galletti C (2010) Cortical connections of parietal field PEc in the macaque: linking vision and somatic sensation for the control of limb action. Cereb Cortex 20:2592–2604. 10.1093/cercor/bhq007 20176687

[B5] Benjamin DJ, et al. (2018) Redefine statistical significance. Nat Hum Behav 2:6–10. 10.1038/s41562-017-0189-z 30980045

[B6] Brown IE, Loeb GE (2000) Measured and modeled properties of mammalian skeletal muscle: IV. Dynamics of activation and deactivation. J Muscle Res Cell Motil 21:33–47. 10.1023/A:1005687416896 10813633

[B7] Carland MA, Marcos E, Thura D, Cisek P (2016) Evidence against perfect integration of sensory information during perceptual decision making. J Neurophysiol 115:915–930. 10.1152/jn.00264.2015 26609110

[B8] Carsten T, Fievez F, Duque J (2023) Movement characteristics impact decision-making and vice versa. Sci Rep 13:3281. 10.1038/s41598-023-30325-4 36841847 PMC9968293

[B9] Česonis J, Franklin DW (2020) Time-to-target simplifies optimal control of visuomotor feedback responses. eNeuro 7:1–17. 10.1523/ENEURO.0514-19.2020 32213555 PMC7189480

[B10] Cisek P, Puskas GA, El-Murr S (2009) Decisions in changing conditions: the urgency-gating model. J Neurosci 29:11560–11571. 10.1523/JNEUROSCI.1844-09.2009 19759303 PMC6665752

[B11] Crevecoeur F, Kurtzer I, Bourke T, Scott SH (2013) Feedback responses rapidly scale with the urgency to correct for external perturbations. J Neurophysiol 110:1323–1332. 10.1152/jn.00216.2013 23825396

[B12] Crevecoeur F, Munoz DP, Scott SH (2016) Dynamic multisensory integration: somatosensory speed trumps visual accuracy during feedback control. J Neurosci 36:8598–8611. 10.1523/JNEUROSCI.0184-16.2016 27535908 PMC6601898

[B13] Cross KP, Cluff T, Takei T, Scott SH (2019) Visual feedback processing of the limb involves two distinct phases. J Neurosci 39:6751–6765. 10.1523/JNEUROSCI.3112-18.2019 31308095 PMC6703887

[B14] Cross KP, Cook DJ, Scott SH (2024) Rapid online corrections for proprioceptive and visual perturbations recruit similar circuits in primary motor cortex. eNeuro 11:1–22. 10.1523/ENEURO.0083-23.2024 38238081 PMC10867723

[B15] Dimitriou M, Wolpert DM, Franklin DW (2013) The temporal evolution of feedback gains rapidly update to task demands. J Neurosci 33:10898–10909. 10.1523/JNEUROSCI.5669-12.2013 23804109 PMC3724995

[B16] Ernst MO, Banks MS (2002) Humans integrate visual and haptic information in a statistically optimal fashion. Nature 415:429–433. 10.1038/415429a 11807554

[B17] Ferrari A, Noppeney U (2021) Attention controls multisensory perception via 2 distinct mechanisms at different levels of the cortical hierarchy. PLoS Biol 19:e3001465. 10.1371/journal.pbio.3001465 34793436 PMC8639080

[B18] Franklin S, Wolpert DM, Franklin DW (2017) Rapid visuomotor feedback gains are tuned to the task dynamics. J Neurophysiol 118:2711–2726. 10.1152/jn.00748.2016 28835530 PMC5672538

[B19] Harris CM, Wolpert DM (1998) Signal-dependent noise determines motor planning. Nature 394:780–784. 10.1038/29528 9723616

[B20] Izawa J, Shadmehr R (2008) On-line processing of uncertain information in visuomotor control. J Neurosci 28:11360–11368. 10.1523/JNEUROSCI.3063-08.2008 18971478 PMC2729125

[B21] Jones KE, Hamilton AF, Wolpert DM (2002) Sources of signal-dependent noise during isometric force production. J Neurophysiol 88:1533–1544. 10.1152/jn.2002.88.3.1533 12205173

[B22] Kasuga S, Crevecoeur F, Cross KP, Balalaie P, Scott SH (2022) Integration of proprioceptive and visual feedback during online control of reaching. J Neurophysiol 127:354–372. 10.1152/jn.00639.2020 34907796 PMC8794063

[B23] Kelly SP, O’Connell RG (2013) Internal and external influences on the rate of sensory evidence accumulation in the human brain. J Neurosci 33:19434–19441. 10.1523/JNEUROSCI.3355-13.2013 24336710 PMC6618757

[B24] Keyser J, Medendorp WP, Oostwoud-Wijdenes L, Selen LPJ (2023) Late integration of vision and proprioception during perturbed reaches. J Neurophysiol 129:1282–1292. 10.1152/jn.00324.2022 37073978

[B25] Körding KP, Wolpert DM (2004) Bayesian integration in sensorimotor learning. Nature 427:244–247. 10.1038/nature02169 14724638

[B26] Lakens D (2013) Calculating and reporting effect sizes to facilitate cumulative science: a practical primer for t-tests and ANOVAs. Front Psychol 4:863. 10.3389/fpsyg.2013.00863 24324449 PMC3840331

[B27] Lakens D, et al. (2018) Justify your alpha. Nat Hum Behav 2:168–171. 10.1038/s41562-018-0311-x

[B28] Maurus P, Jackson K, Cashaback JGA, Cluff T (2023) The nervous system tunes sensorimotor gains when reaching in variable mechanical environments. iScience 26:106756. 10.1016/j.isci.2023.106756 37213228 PMC10197011

[B29] Murphy PR, Boonstra E, Nieuwenhuis S (2016) Global gain modulation generates time-dependent urgency during perceptual choice in humans. Nat Commun 7:13526. 10.1038/ncomms13526 27882927 PMC5123079

[B30] Nashed JY, Crevecoeur F, Scott SH (2012) Influence of the behavioral goal and environmental obstacles on rapid feedback responses. J Neurophysiol 108:999–1009. 10.1152/jn.01089.2011 22623483

[B31] Oldfield RC (1971) The assessment and analysis of handedness: the Edinburgh inventory. Neuropsychologia 9:97–113. 10.1016/0028-3932(71)90067-4 5146491

[B32] Oostwoud Wijdenes L, Brenner E, Smeets JBJ (2011) Fast and fine-tuned corrections when the target of a hand movement is displaced. Exp Brain Res 214:453–462. 10.1007/s00221-011-2843-4 21874536 PMC3178780

[B33] Oostwoud Wijdenes L, Medendorp WP (2017) State estimation for early feedback responses in reaching: intramodal or multimodal? Front Integr Neurosci 11:38. 10.3389/fnint.2017.00038 29311860 PMC5742230

[B34] Poscente SV, Peters RM, Cashaback JGA, Cluff T (2021) Rapid feedback responses parallel the urgency of voluntary reaching movements. Neuroscience 475:163–184. 10.1016/j.neuroscience.2021.07.014 34302907

[B35] Prat-Ortega G, Wimmer K, Roxin A, de la Rocha J (2021) Flexible categorization in perceptual decision making. Nat Commun 12:1283. 10.1038/s41467-021-21501-z 33627643 PMC7904789

[B36] Pruszynski AJ, King GL, Boisse L, Scott SH, Flanagan JR, Munoz DP (2010) Stimulus-locked responses on human arm muscles reveal a rapid neural pathway linking visual input to arm motor output. Eur J Neurosci 32:1049–1057. 10.1111/j.1460-9568.2010.07380.x 20726884

[B37] Ratcliff R (1978) A theory of memory retrieval. Psychol Rev 8:59–108. 10.1037/0033-295X.85.2.59 3406246

[B38] Ratcliff R, Smith PL, Brown SD, McKoon G (2016) Diffusion decision model: current issues and history. Trends Cogn Sci 20:260–281. 10.1016/j.tics.2016.01.007 26952739 PMC4928591

[B39] Reddi BAJ, Carpenter RHS (2000) The influence of urgency on decision time. Nat Neurosci 3:827–830. 10.1038/77739 10903577

[B40] Resulaj A, Kiani R, Wolpert DM, Shadlen MN (2009) Changes of mind in decision-making. Nature 461:263–266. 10.1038/nature08275 19693010 PMC2875179

[B41] Scott SH (2016) A functional taxonomy of bottom-up sensory feedback processing for motor actions. Trends Neurosci 39:512–526. 10.1016/j.tins.2016.06.001 27378546

[B42] Selen LPJ, Shadlen MN, Wolpert DM (2012) Deliberation in the motor system: reflex gains track evolving evidence leading to a decision. J Neurosci 32:2276–2286. 10.1523/JNEUROSCI.5273-11.2012 22396403 PMC3299561

[B43] Stanford TR, Salinas E (2021) Urgent decision making: resolving visuomotor interactions at high temporal resolution. Annu Rev Vis Sci 7:323–348. 10.1146/annurev-vision-100419-103842 34171199

[B44] Tassinari H, Hudson TE, Landy MS (2006) Combining priors and noisy visual cues in a rapid pointing task. J Neurosci 26:10154–10163. 10.1523/JNEUROSCI.2779-06.2006 17021171 PMC6674625

[B45] Thobois S, Ballanger B, Baraduc P, Le Bars D, Lavenne F, Broussolle E, Desmurget M (2007) Functional anatomy of motor urgency. Neuroimage 37:243–252. 10.1016/j.neuroimage.2007.04.049 17553705

[B46] Thura D, Beauregard-Racine J, Fradet CW, Cisek P (2012) Decision making by urgency gating: theory and experimental support. J Neurophysiol 108:2912–2930. 10.1152/jn.01071.2011 22993260

[B47] Thura D, Cabana JF, Feghaly A, Cisek P (2022) Integrated neural dynamics of sensorimotor decisions and actions. PLoS Biol 20:e3001861. 10.1371/journal.pbio.3001861 36520685 PMC9754259

[B48] Todorov E (2005) Stochastic optimal control and estimation methods adapted to the noise characteristics of the sensorimotor system. Neural Comput 17:1084–1108. 10.1162/0899766053491887 15829101 PMC1550971

[B49] Todorov E, Jordan MI (2002) Optimal feedback control as a theory of motor coordination. Nat Neurosci 5:1226–1235. 10.1038/nn963 12404008

[B50] Tsay JS, Avraham G, Kim HE, Parvin DE, Wang Z, Ivry RB (2021) The effect of visual uncertainty on implicit motor adaptation. J Neurophysiol 125:12–22. 10.1152/jn.00493.2020 33236937 PMC8087384

[B51] Van Beers RJ, Sittig AC, Denier Van Der Gon JJ (1996) How humans combine simultaneous proprioceptive and visual position information. Exp Brain Res 111:253–261. 10.1007/BF00227302 8891655

[B52] Visser YF, Medendorp WP, Selen LPJ (2023) Muscular reflex gains reflect changes of mind in reaching. J Neurophysiol 130:640–651. 10.1152/jn.00197.2023 37584102

[B53] Wolpert DM, Landy MS (2012) Motor control is decision-making. Curr Opin Neurobiol 22:996–1003. 10.1016/j.conb.2012.05.003 22647641 PMC3434279

