# Supplementary figures and images for: Dissociable Effects of Urgency and Evidence Accumulation during Reaching Revealed by Dynamic Multisensory Integration
Source: eNeuro. 2024 Dec 4;11(12):ENEURO.0262-24.2024. doi: 10.1523/ENEURO.0262-24.2024 (PMC11628215; doi:10.1523/ENEURO.0262-24.2024)

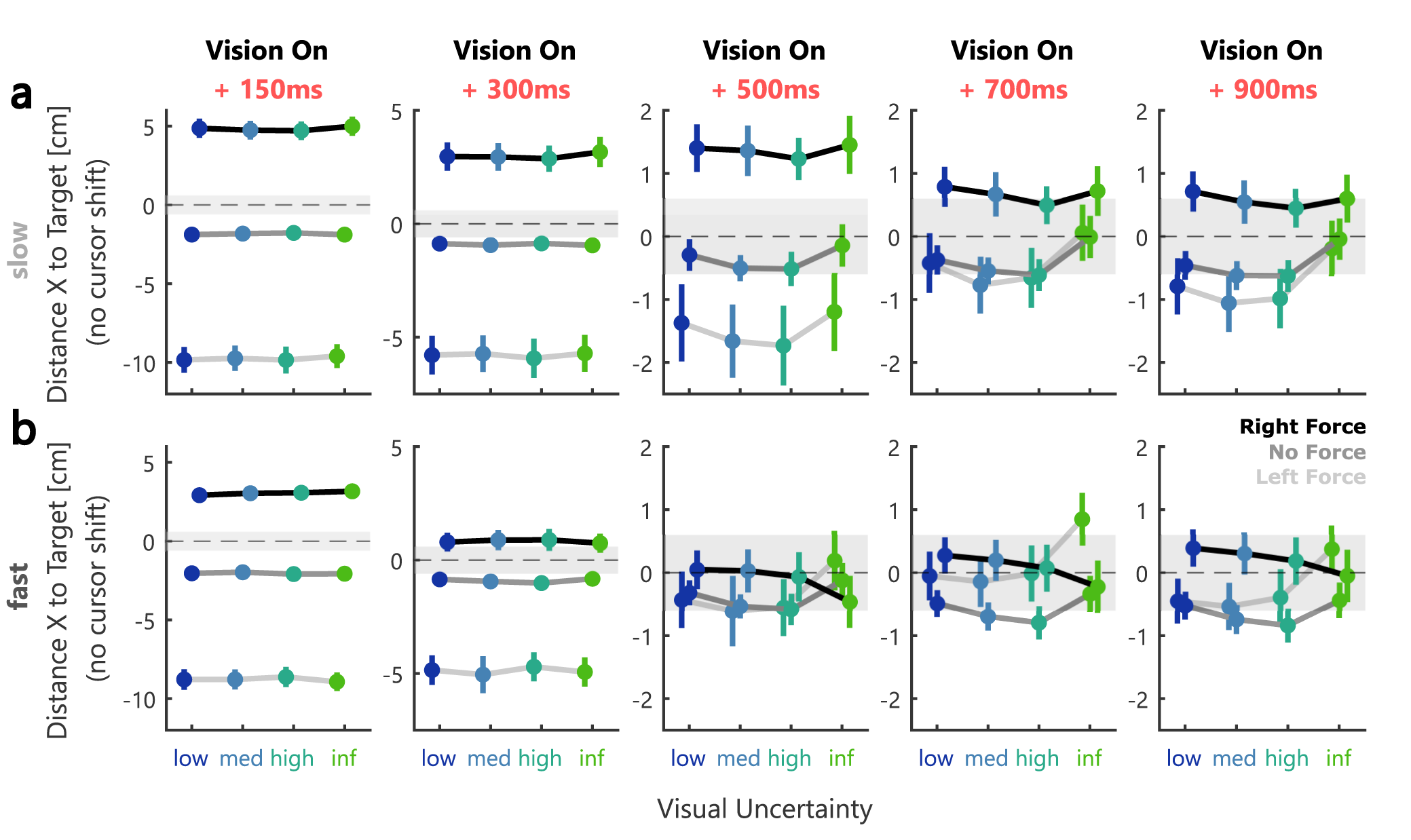

Supplement: Figure 3-1 — Lateral offset relative to the target at the end of the movement does not vary systematically with visual uncertainty or movement speed. (a) Lateral (x-dimension) offset between the hand and the target midline at different timepoints following the onset of the visual feedback during trials without cursor shift in the slow movement condition. Lines in different shades of gray indicate the different force perturbation directions (black = rightward force, dark gray = no force, light gray = leftward force). The dashed horizontal line and the light gray shaded region mark the center and diameter of the target circle, respectively. (b) Same as (a) but for the fast movement condition. Download Figure 3-1, TIF file. [file eneuro-11-ENEURO.0262-24.2024-s001.tif]

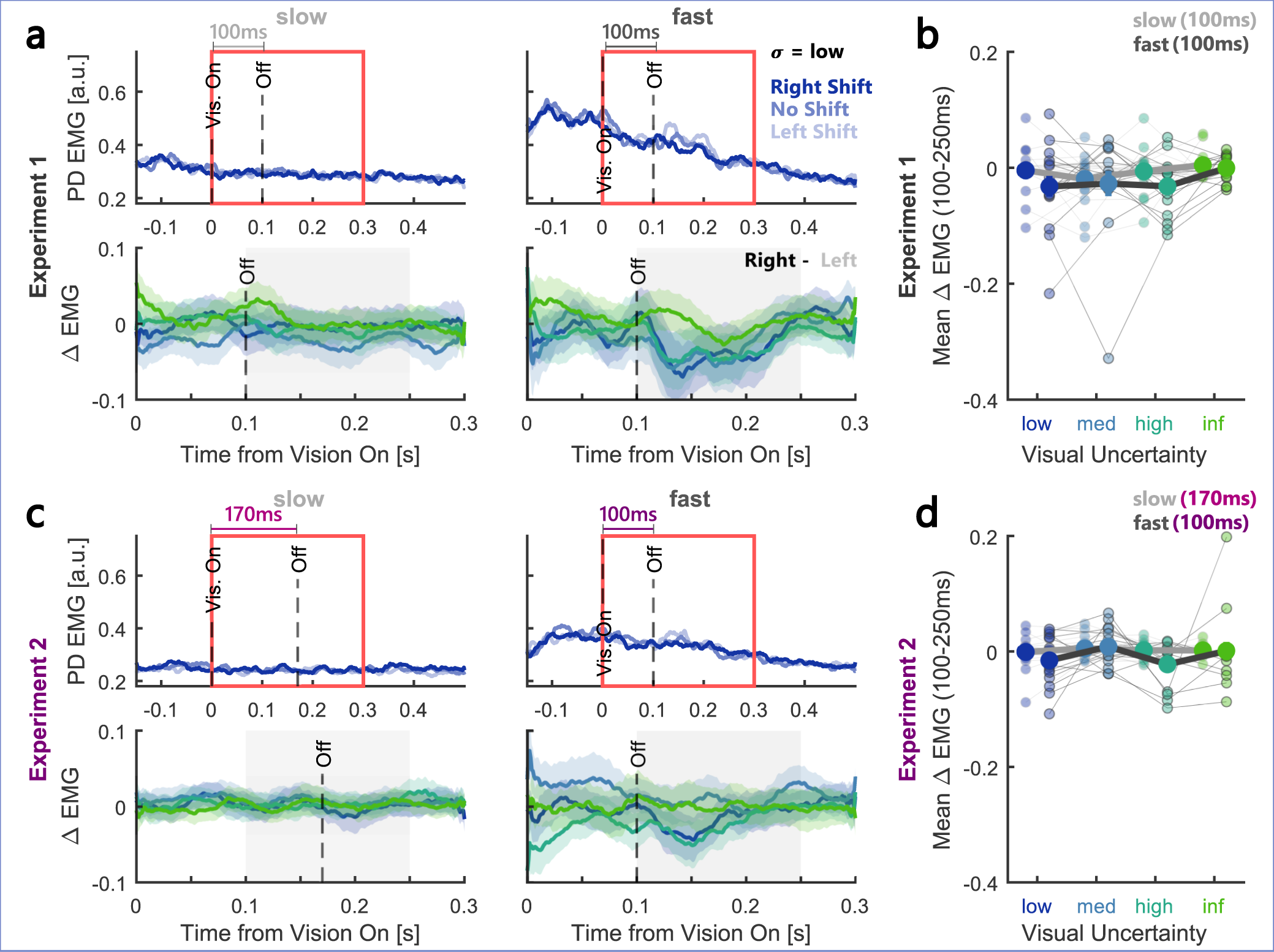

Supplement: Figure 6-1 — Antagonist muscle activity does not vary with visual feedback. (a) Top: Group average posterior deltoid EMG activity during rightward force perturbations and low visual uncertainty in the slow (left) and fast (right) movement condition in experiment 1. Data was aligned to the onset time of the visual feedback. Shades of blue correspond to the three different cursor shift conditions. Dashed black lines indicate onset and offset of visual feedback. Bottom: Zoom-in of the difference in EMGs to rightward - leftward cursor shift during slow (left) and fast (right) movements in experiment 1. The area that is zoomed-in is depicted by a red box in the top panels. In experiment 1 the visual feedback duration was 100 ms for both slow and fast conditions as indicated by the gray/black time bars in the top panels. (b) EMG responses averaged across 100-250 ms following vision onset (gray-shaded area shown in (a) bottom panels) as a function of visual uncertainty. The slow condition is represented by light gray lines, the fast condition by dark gray lines. Group averages are shown as thick lines and individual participants’ data as thin lines. (c) Same as (a) but for experiment 2. Note that in experiment 2, the slow movement condition had a visual feedback duration of 170 ms while the fast movement condition was identical to experiment 1 (100 ms feedback duration) as indicated by the pink/purple time bars in the top panels. (d) Same as (b) but for experiment 2. The color-coding is identical to the one used in figure 2 and the error-bars indicate group-averages ± SEM. Download Figure 6-1, TIF file. [file eneuro-11-ENEURO.0262-24.2024-s002.tif]

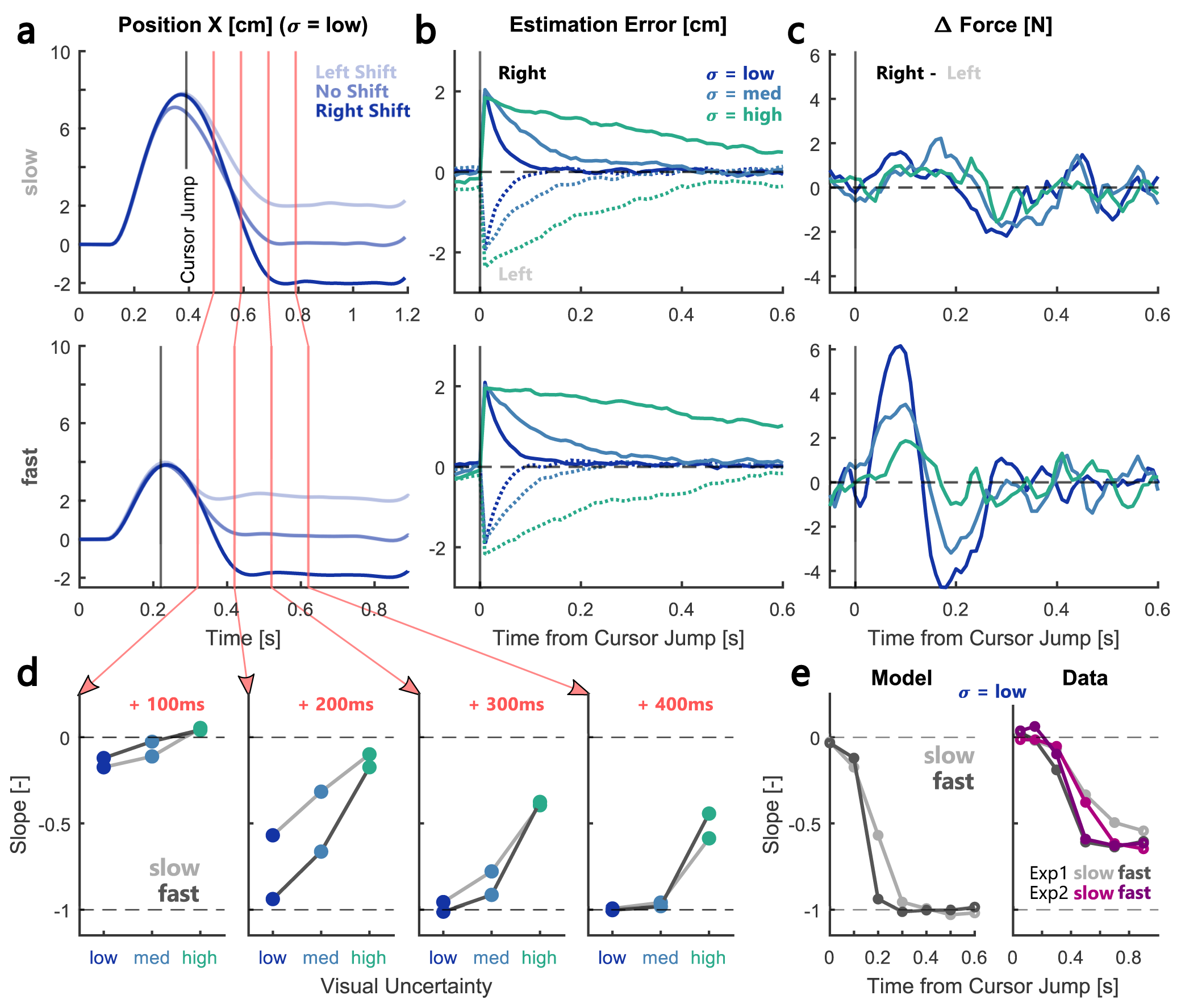

Supplement: Figure 8-1 — Model simulations with signal-dependent noise also reproduce effect of visual uncertainty and movement speed. (a) Simulated lateral positions over time during slow (top) and fast (bottom) movements with low visual uncertainty. The black line marks the moment the cursor jump was applied and the shades of blue correspond to the direction of the jump. Red vertical lines correspond to 100, 200, 300, and 400 ms after the cursor jump. (b) Average decay of the estimation error following the cursor jump during different visual uncertainties (blues & greens). The decay following a leftward cursor jump is shown as dotted lines, that following rightward jumps as full lines. (c) Difference in lateral forces to rightward - leftward cursor jumps during slow (top) and fast (bottom) simulated movements. (d) Slopes of the simulated movements at the four highlighted timepoints following the cursor jump as a function of visual uncertainty. Slow movements are shown as light gray lines and fast as dark gray lines. (e) The development of average slopes during low visual uncertainty over time from cursor jump/vision onset. Simulated data is shown on the left and experimental data on the right. Data of experiment 1 is plotted in gray and data from experiment 2 in pink/purple. Refer to Extended data 1 for the model code used to generate this figure. Download Figure 8-1, TIF file. [file eneuro-11-ENEURO.0262-24.2024-s003.tif]

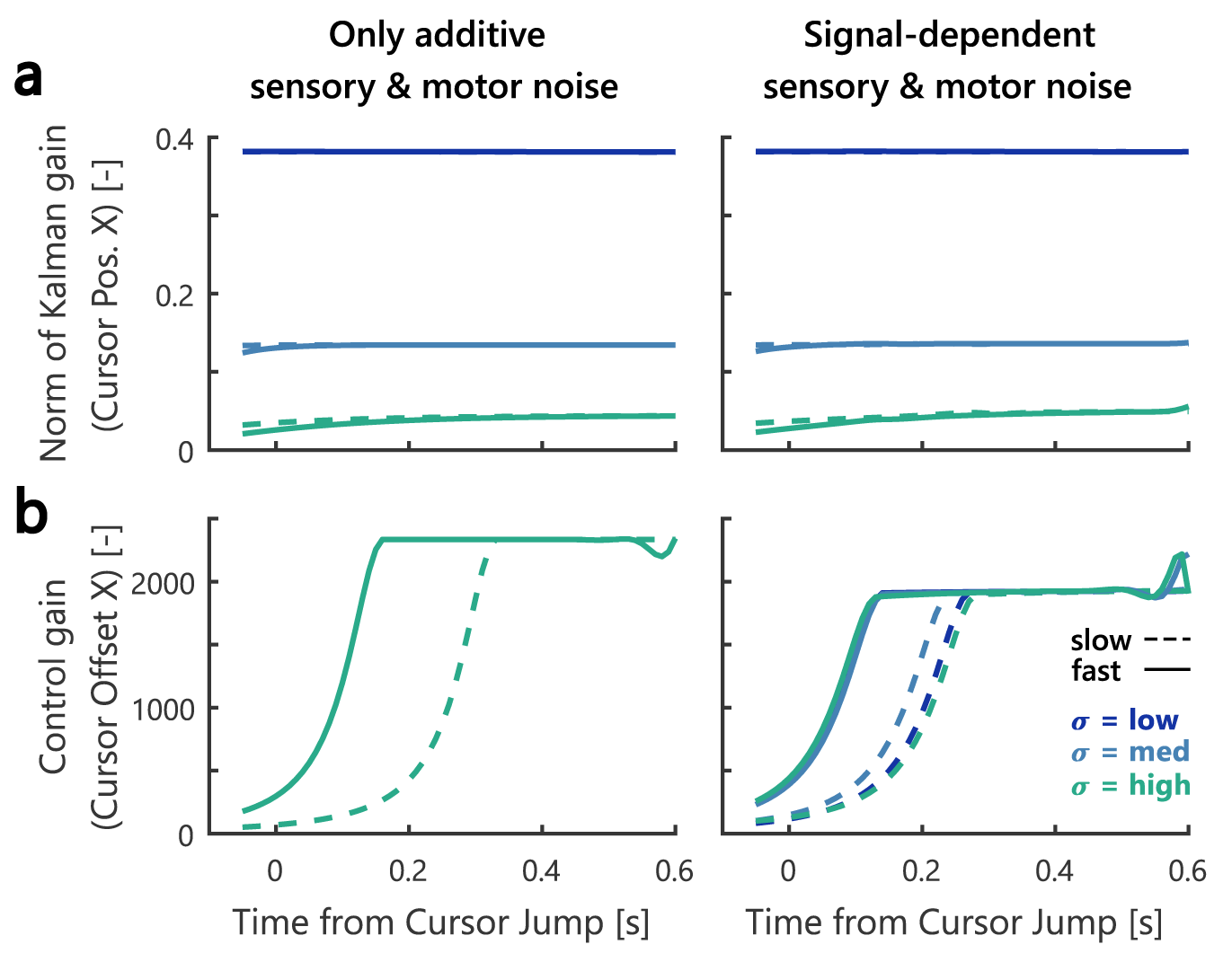

Supplement: Figure 8-2 — Comparison of Kalman and control gains between models without and with signal-dependent noise. (a) Effect of visual sensory noise and movement speed on the norm of the block components of the Kalman gain matrix (Eq. 6) influencing the estimation of the lateral (x-dimension) position of the visual cursor. Data was aligned to the onset of the cursor jump in the simulations. Blue-green colors indicate different visual uncertainty levels. Dashed lines show values for slow movements and full lines those for fast movements. (b) Effect of visual sensory noise and movement speed on the control gains (Eq. 8) corresponding to the lateral offset between the simulated point mass and the cursor. The alignment and color-coding are the same as in (a). Refer to Extended data 1 for the model code used to generate this figure. Download Figure 8-2, TIF file. [file eneuro-11-ENEURO.0262-24.2024-s004.tif]
